# Non-Invasive Brain-Computer Interfaces: State of the Art and Trends

**DOI:** 10.1109/RBME.2024.3449790

**Published:** 2025-01-28

**Authors:** Bradley J. Edelman, Shuailei Zhang, Gerwin Schalk, Peter Brunner, Gernot Müller-Putz, Cuntai Guan, Bin He

**Affiliations:** Max Planck Institute of Psychiatry, 80804 Munich, Germany; Max Planck Institute for Biological Intelligence, 82152 Planegg, Germany.; Nanyang Technological University, Singapore 639798.; Fudan University, Shanghai 200433, China.; Washington University in St. Louis, St. Louis, MO 63130-4899 USA.; Graz University of Technology, A-8010 Graz, Austria.; Nanyang Technological University, Singapore 639798.; Carnegie Mellon University, Pittsburgh, PA 15217 USA

**Keywords:** BCI, brain-computer interface, deep learning, electroencephalography, manifold classification, motor imagery, motor-related cortical potentials, neural decoding, neurotechnology, robotic arm, transfer learning

## Abstract

Brain-computer interface (BCI) is a rapidly evolving technology that has the potential to widely influence research, clinical and recreational use. Non-invasive BCI approaches are particularly common as they can impact a large number of participants safely and at a relatively low cost. Where traditional non-invasive BCIs were used for simple computer cursor tasks, it is now increasingly common for these systems to control robotic devices for complex tasks that may be useful in daily life. In this review, we provide an overview of the general BCI framework as well as the various methods that can be used to record neural activity, extract signals of interest, and decode brain states. In this context, we summarize the current state-of-the-art of non-invasive BCI research, focusing on trends in both the application of BCIs for controlling external devices and algorithm development to optimize their use. We also discuss various open-source BCI toolboxes and software, and describe their impact on the field at large.

## I. Introduction

A BRAIN-computer interface (BCI) is a neurotechnology that enables direct brain-based communication between an individual and the rest of the world [1], [2]. While BCIs could have broad applications in various patient cohorts and even the general population, they have specifically targeted users suffering from neuromuscular impairments such as amyotrophic lateral sclerosis (ALS) [3], [4], [5], spinal cord injury (SCI) [6], [7], [8], or stroke [9], [10], [11], but whose cognitive function remains intact. Indeed, clinical applications of BCIs, while still limited to research use, have become more accepted than ever and often complement other therapeutic strategies [10], [12], [13], [14]. This technology has also recently gained recreational popularity (e.g., “brain games”) [15], [16] as neural recordings become increasingly accessible due to low-cost systems and open-source toolboxes. As such, the concept of human enhancement and restoration through brain-controlled channels now allows individuals to connect with others on a level not previously achievable.

In its most basic form, a BCI is a system that decodes the user’s mental state or intention and maps such information to the action of a device that interacts with the surrounding environment [17], [18]. These interactions can convey information that range from answering simple yes/no questions, to constructing word-based communication, to the navigation and control of devices such as robotic arms, wheelchairs, and more. Nevertheless, the widespread research space of BCI has also facilitated new and creative applications for this technology. For example, rather than decoding an intended sentence from a word or letter bank presented on a computer screen, modern BCIs can now decode handwriting signals directly from the brain [19]. In addition, extra-sensory BCIs have been proposed that detect and in some cases provide closed-loop feedback to alter various affective states [20], [21], [22]. Other interesting avenues of BCIs investigate how sensory feedback, whether provided to the peripheral (i.e., haptic feedback) [23], [24] or central (i.e., intracortical micro-stimulation [25], transcranial electrical stimulation [26], or focused ultrasound stimulation [27]) nervous system, impacts the performance of these systems for completing various tasks.

While these examples represent exciting and innovative avenues for the field of BCI, the direction of BCI subfields is often guided by how easily and robustly mental intention can be decoded from brain recordings. As such, there are various methods available to acquire neural signals, including both non-invasive and invasive approaches that exhibit different signal-to-noise ratios, spatial coverage, spatiotemporal resolution, etc. This spectrum of signal acquisition techniques is accompanied by a broad collection of mental states and tasks that can be incorporated into the BCI system. For invasive BCIs, high-resolution recordings provide information regarding detailed brain states and dynamics (often related to motor function), however, these systems are often constrained to a limited number of clinical participants or basic research in rodents and non-human primates (NHPs) [28], [29]. Non-invasive BCIs, on the other hand, minimally impact the user without safety concerns, are easy to use in everyday life, and facilitate long-term performance tracking in a large number of participants. Nevertheless, current non-invasive BCIs exhibit limited performance due to the limited signal-to-noise ratio and information transfer rate that occurs when neural signals transmit from brain tissues, through the skull, and to the scalp. The easy access to many suitable participants, however, offers opportunities that enable non-invasive BCI researchers to push the limits of robust paradigms and decoding techniques to optimize task accuracy, information transfer, and system reliability for human applications.

In the current review, we focus on recent innovations in the field of non-invasive BCI research. We first recount traditional BCI signal acquisition methods and describe various new approaches to measuring information from the brain. We then provide detailed accounts of different non-invasive signal types and highlight how these signals have been utilized to achieve the current state-of-the-art demonstrations of brain-based device control. We further outline various relevant algorithmic trends that enable high-performance EEG-based brain state decoding. Finally, we review different BCI toolboxes and software that are available to aid in performing reliable BCI experiments at a large scale.

## II. Invasive and Non-Invasive BCI

**Fig. 1.**
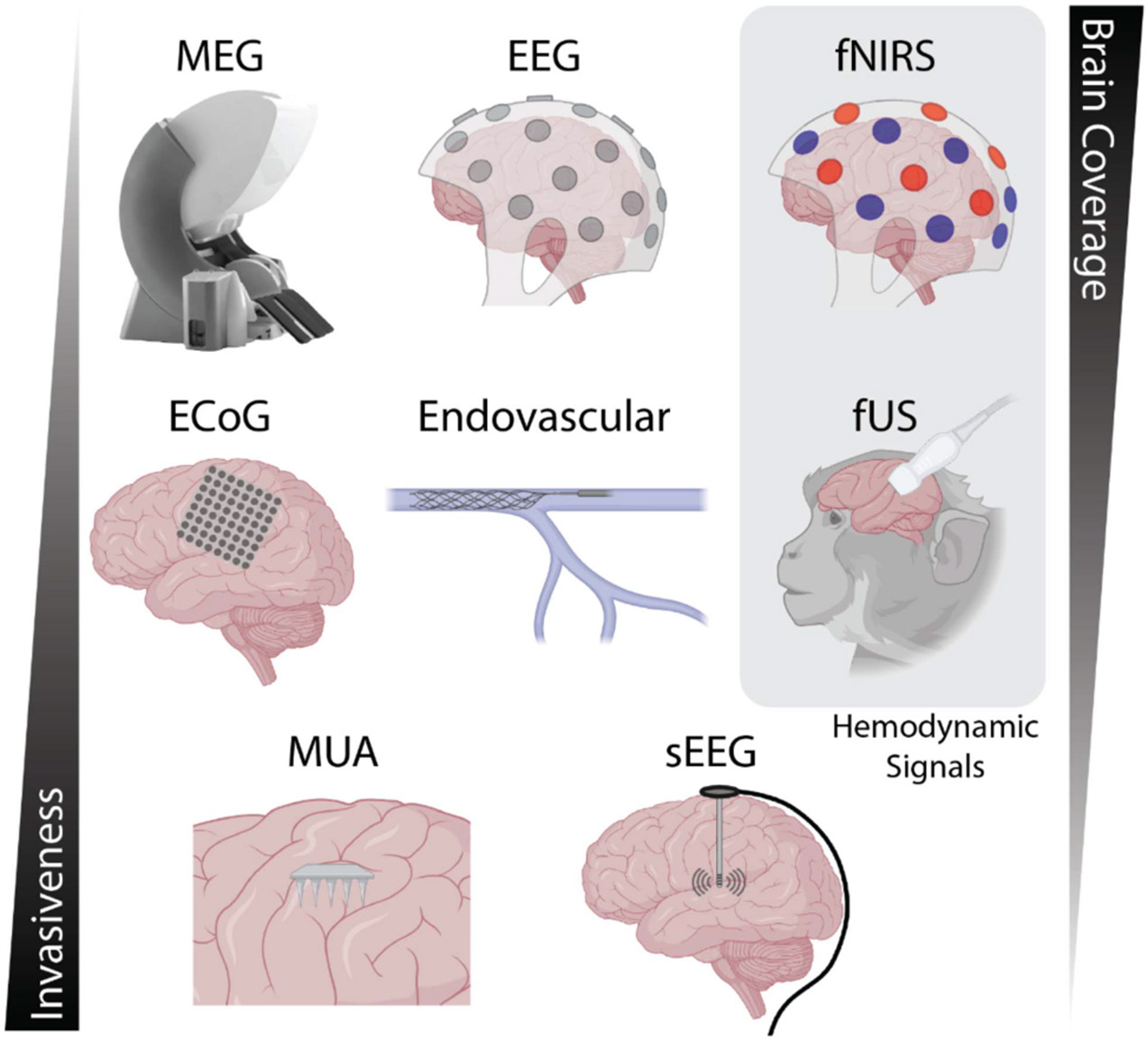
Neural recording modalities used for brain-computer interface applications. (top) Non-invasive techniques include magnetoencephalography (MEG), electroencephalography (EEG) and functional near-infrared spectroscopy (fNIRS). (middle) Minimally invasive approaches involve electrocorticography (ECoG) and relatively novel technologies such as endovascular electrodes (i.e., the Stentrode) and functional ultrasound (fUS). (bottom) Invasive techniques include stereoEEG (sEEG) with penetrating electrodes and multi-unit arrays (i.e., the Utah Array). Nearly all these techniques measure electrophysiological signals with the exception of fNIRS and fUS, which measure an indirect hemodynamic readout of neuronal activity.

All BCIs have the common goal of decoding an individual’s mental state or intention. However, there exists an implicit dichotomy between invasive and non-invasive BCI technology. A middle ground of minimally invasive techniques that require direct brain access but that do not penetrate neural tissue has also shown promise (Fig. 1). In general, there are notable differences in the complexity of deploying these various techniques, many of which also provide varying levels of brain coverage and signal quality. Currently, invasive BCI technology is limited to patients enrolled in clinical trials and the use thereof involves a lengthy timeline that encompasses surgical planning and task training. While invasive BCIs reach a limited number of individuals, the detail of control has seen significant advancements due to the high-fidelity signals that are available. In this regard, notable achievements include the control of robotic arms and lower limb exoskeletons, speech decoding, and more.

Invasive BCIs that leverage motor-related signals (e.g., motor cortex, posterior parietal cortex, etc.) have traditionally utilized Utah arrays or other multi-site recording probes. These arrays provide detailed multi-unit activity (MUA) from hundreds of recording sites and have enabled the control of robotic arms, patients’ own arms, and speech decoding [30], [31], [32], [33], [34]. However, chronic use of these electrodes is hindered by a lack of long-term signal quality and the high power requirements needed for high temporal sampling of the signals of interest. As such, invasive recording methods often require physically wired connections between the brain implant and the decoding hardware, power supply, etc. Therefore, in addition to the need for surgical implantation of the recording device, these technical constraints limit widespread clinical use. Nevertheless, there are ongoing attempts using both lower fidelity signals and more advanced silicone electrodes (i.e., Neuropixels) that may, in the future, enable wireless systems that can be utilized in a larger patient population [35], [36]. Stereo-electroencephalography (sEEG) is an alternative invasive technique that utilizes penetrating electrodes inserted into deep regions of the brain. These electrodes are typically used for monitoring brain pathologies such as epilepsy but are also gaining recognition for closed-loop stimulation [22] and other BCI applications [37].

An alternative to these invasive and penetrating electrode arrays is minimally invasive electrocorticographic (ECoG) grids that record neural activity from the surface of the brain [8], [38]. This approach covers a larger area of the brain than penetrating arrays, records lower fidelity signals, and enables wireless data transfer. ECoG-based BCIs have long shown promise for clinical use, but were often limited to individuals undergoing temporary brain monitoring when a craniotomy was required for other purposes. However, the introduction of self-contained devices such as the WIMAGINE system [39], which consists of an implantable ECoG grid that can be stably mounted to the skull, has been useful for BCI-specific purposes. In fact, using the WIMAGINE device has gained notoriety for facilitating the restoration of walking ability by combining motor-related brain signals with either robotic exoskeletons or spinal cord stimulation [40], [41]. These individual demonstrations have shown significant clinical BCI translation, and the similarity in signal characteristics between ECoG and EEG gives rise to the question of when and if similar achievements can be made completely non-invasively.

Within the past few years, the field of BCI has also seen the rapid rise of two relatively novel approaches to minimally invasive neural recordings. One promising approach to recording signals from the brain is through the use of an endovascular electrode placed within the lumen of large cortical vessels such as the superior sagittal sinus [42]. This type of electrode, termed the Stentrode, resembles a simple stent that also contains several recording/stimulation sites and can be inserted into the brain using standard endovascular medical procedures. However, as this device relies on vessel location in the brain, the recording coverage is limited to tissue within the cleft between the two brain hemispheres, often targeting contact with the precentral gyrus. With the topographic organization of the motor homunculus, these electrodes primarily detect motor commands related to the feet, but may, in the future, incorporate other mental states as well. Nevertheless, the Stentrode received approval for human BCI use from the United States Federal Drug Administration (FDA) and has already shown successful control of computer cursor-related tasks in patients [43], [44].

Functional ultrasound (fUS) has also recently demonstrated feasibility in decoding motor intentions directly from the brain. fUS measures an indirect hemodynamic readout of neuronal activity at a relatively slow temporal resolution (~2–10 Hz) and requires a cranial window and/or an acoustically transparent skull prosthetic for high-quality signal acquisition [45], [46]. Despite the need for this procedure, fUS offers a potential alternative to chronic electrical recordings that can degrade over time. The use of this technology for BCI applications is still in its infancy, but has already demonstrated the presence of discriminable motor intention signals in the parietal cortex in NHPs that can be detected in real-time [47], [48]. Importantly, fUS has recently also been used to record motor-related signals in a patient with a sonolucent cranial implant [46], indicating that human BCI applications are on the horizon. Nevertheless, transcranial fUS has yet to be realized in healthy adult individuals or patients at a spatio-temporal resolution (and without contrast agents) that would benefit large-scale BCI studies. While matrix mixing and aberration correction approaches [49], [50] show promise for transcranial fUS imaging, these concepts need to be further validated *in vivo*.

Non-invasive BCI technology primarily utilizes EEG recordings that measure electrical potentials from the scalp. Other approaches utilize magnetoencephalography (MEG) or functional near-infrared spectroscopy (fNIRS) that respectively measure magnetic and hemodynamic information from the brain, however, these are less popular. As EEG is cheap, completely non-invasive, easy to set up, and temporary, it can be employed by large studies with as many as thousands of human participants to obtain robust statistical conclusions [51], [52]. Furthermore, non-invasive recording modalities uniquely record whole-brain activity and provide a variety of signal types that can be used individually or in a hybrid fashion to construct a BCI. These signal types (as described in the next section) are often robust and can be extracted for BCI purposes using minimal processing steps. The ease-of-use and low barrier-to-entry have, in turn, led to more creative BCI applications for both research and consumer applications that incorporate mobile use. As such, there are now dozens of wireless and/or dry electrode EEG systems that enable the acquisition of up to 128 channels at > 2 kHz [53], [54]. Importantly, wireless systems avoid artifacts associated with cable movement and facilitate use in ambulatory tasks that will eventually be required to perform neural recordings in naturalistic settings. However, mobile tasks and locomotion also introduce other distortions related to the gait cycle and head movement that must be further characterized and accounted for [55], [56].

## III. Non-Invasive BCI Signals

**Fig. 2.**
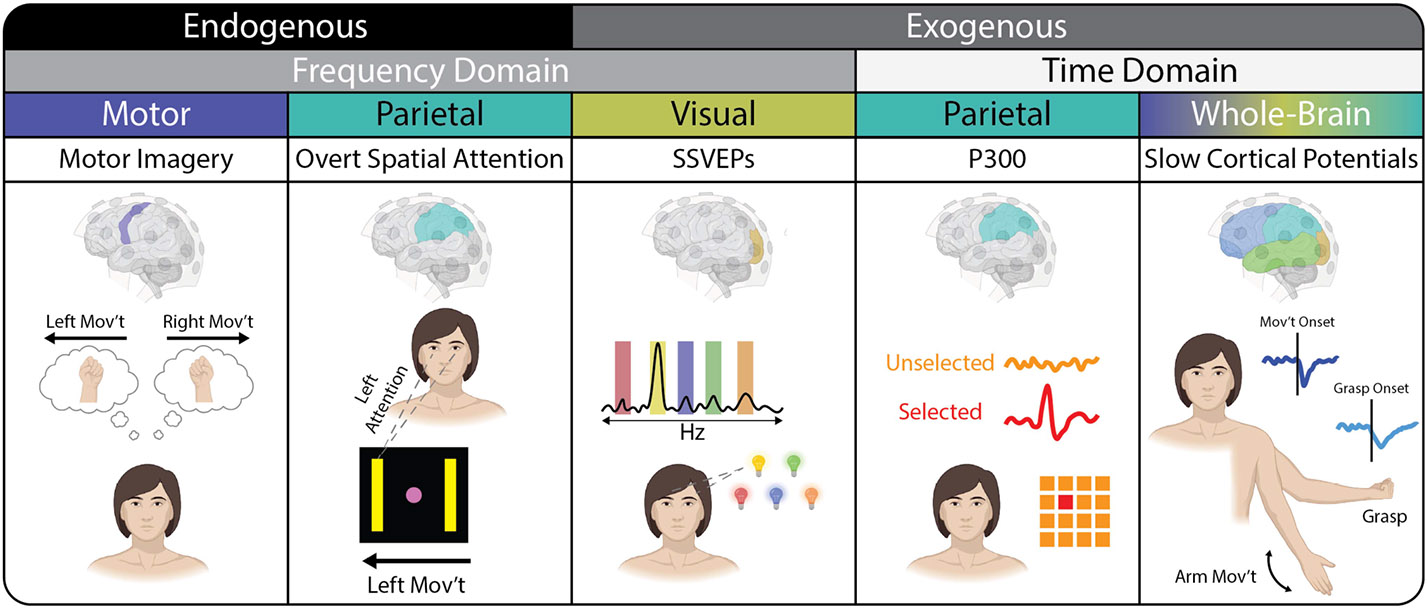
Overview of neural signals used for noninvasive brain-computer interface control. These signals can be broadly categorized according to being endogenous or exogenous in origin, frequency or time domain, and by the brain region/electrode coverage from which they are acquired. Motor imagery tasks generate an event-related (de)synchronization that can be detected from motor electrodes and which generate a velocity-based signal for end effector control direction. Overt spatial attention refers to a gaze-based action that is detected in electrodes covering the parietal cortex and which drives an end effector in a particular direction. Steady-state visual evoked potentials (SSVEPs) refer to increases in narrow band power in electrodes covering the visual cortices upon attending to a stimulus flickering at the corresponding frequency. The P300 response is elicited during an oddball-type context when a user’s choice is selected. Finally, slow cortical potentials refer to electrical potential deflections that are time-locked to movement events or that correspond to limb kinematics.

For the remainder of this review we discuss various types of signals used for non-invasive BCIs (see Fig. 2).

### A. Visual Evoked Potentials

Visual evoked potentials (VEPs) rely on the conscious recognition of a target in the user’s visual field. VEPs depend on external stimuli, which generally consist of lights flickering with different temporal profiles. These profiles fall into five broad categories: 1) frequency modulation (f-VEP), 2) time modulation (t-VEP), 3) code modulation (c-VEP), 4) phase modulation (p-VEP), and 5) motion-onset (m-VEP). In all of these cases, each stimulus carries a unique temporal pattern according to the modulation scheme that can also be detected in the scalp EEG to indicate the selected stimulus. For example, in the case of t-VEPs and c-VEPs, different stimuli are presented at varying delays from trial onset, which can then be detected in the time-domain EEG signal [57], [58], [59]. However, due to noisy time-domain signals, these VEP variations require a high number of trial averages for robust results. By contrast, f-VEP and p-VEP stimuli flicker at different frequencies and/or phases. Attending to a particular target increases the band power of the corresponding oscillation in electrodes covering the occipital cortex. Thus, f-VEPs and p-VEPs are collectively also known as steady-state VEPs (SSVEPs). In practice, SSVEP-based BCIs provide some of the highest information transfer rates as the combination of frequency and phase modulation can be decoded with high accuracy [60]. This combination also now allows for the successful detection of >100 target options [61], [62], which can be further expanded in hybrid systems when complementary signal types are utilized [63]. Finally, the m-VEP stimuli consist of moving lines across a virtual keyboard to induce visual motion-based event-related potentials [64] to form a BCI speller. In particular, the N200 component of these evoked signals is considered to be primarily related to motion-onset visual processing. These fast and high-dimensional BCIs work well with a variety of applications, including spellers and, less often, spatial navigation, where individual commands need to be correctly identified during relatively narrow time windows.

### B. Overt Spatial Attention

Overt spatial attention (OSA) tasks leverage activity patterns generated in the parietal cortex in response to the user’s spatial attention. Similar to motor imagery (MI) tasks, OSA signals do not require external stimuli and generate focal patterns of alpha band activity. This makes OSA tasks well suited to complement other endogenous signals, such as those generated from performing MI tasks [65], [66], by increasing control dimensions without sacrificing overall performance of the BCI. While this relatively new BCI paradigm shows promise for higher dimensional control, users and experimenters must still consider the interaction effects of overt gaze control and other BCI-related tasks [67], [68].

### C. P300

The P300 is an endogenous event-related potential (ERP) within the EEG, and is detected in electrodes covering the parietal cortex. P300 signals occur in the context of an oddball paradigm [69], and rely on a user’s implicit ability to distinguish a rarely presented target stimulus from other more common non-target stimuli. This structure makes such an event-related response useful for spelling applications where a specific letter must be chosen from a larger set of irrelevant letters. Most P300-based BCIs use the visual row/column paradigm, in which a matrix (e.g., 6 × 6 cells or variable) containing the alphabet, numbers, and other items is presented to the user for selection [70], [71]. A P300 response is then elicited when the rows and columns of the matrix, flashing in random order, converge on the desired item being attended to. The P300 was documented well before BCI use and has long been implicated as a biomarker of various clinical disorders, including depression [72] and schizophrenia [73]. While these signals can be gaze- and space-dependent, advances in stimulus design such as variations of the rapid serial visual presentation approach have significantly improved information transfer rates as well as task reliability and performance [74], [75].

### D. Movement-Related Cortical Potentials

Other broadband time-domain strategies that are favored for the identification of both cued and continuous mental states involve slow cortical potentials, otherwise known as movement-related cortical potentials (MRCPs). When closely examining voluntary movement, changes in low-frequency activity (delta band, ~1 – 4 Hz) occur in the movement planning and execution stages and are time- and phase-locked to movement onset [76], [77]. After averaging across numerous trials, a so-called MRCP becomes visible; the main components are the “Bereitschaftspotential” or readiness potential, the motor potential (negative peak), and the positive rebound potential. It has been shown in the literature that such an MRCP occurs after different voluntary actions and can be derived from sensorimotor areas. Over the last decade, MRCP features have been heavily exploited for decoding movement detection [78], [79], [80], [81], [82], [83], [84], but also for the classification of movement-related parameters such as speed [85] and force [79].

While the success rate of the MRCP-based motor control is modest compared to some other signal types, the burden of imagining different types of movement is dramatically reduced. For example, during the MoreGrasp project [86], study participants described a low burden during the calibration and use of the BCI, suggesting that a successful transfer of MRCPs towards natural control can be achieved. Nevertheless, the false positive and success rates can still be improved, which is a matter of ongoing and future work (see [87] for a comprehensive review).

### E. Event-Related (De)Synchronization

**Fig. 3.**
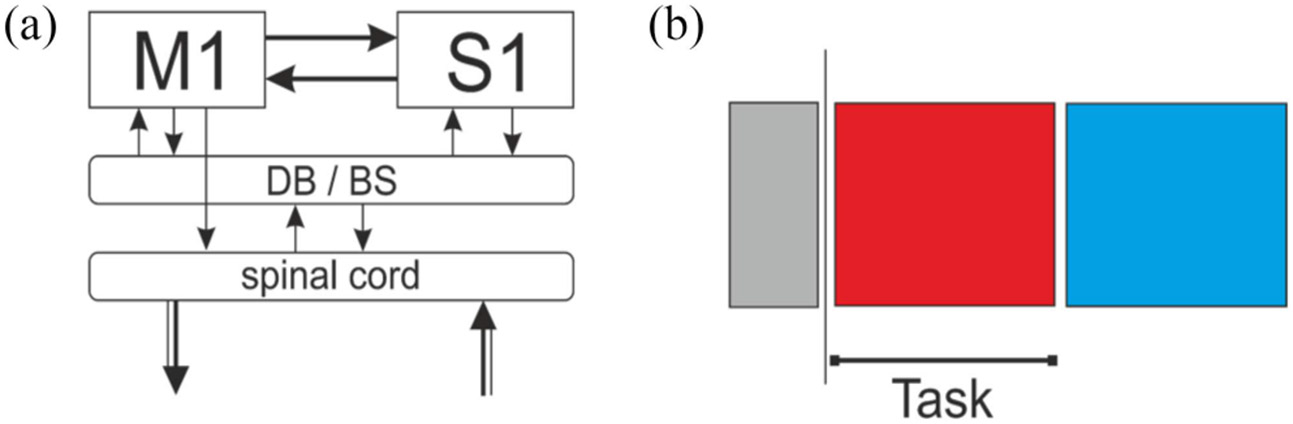
Neuro-structural model and general trial structure to elicit ERD/ERS. a) Neurostructural model for the description and interpretation of resulting EEG patterns. M1 and S1 symbolize primary motor and somatosensory cortex, respectively, and both carry bidirectional pathways to the deep brain and brainstem (DB/BS). M1 projects directly to the spinal cord, which is also connected to the DB/BS via ascending and descending fibers. Efferent and afferent connections in the peripheral nervous system are indicated by double lined arrows. b) The general structure of a task trial consists of a preparation phase (grey box), followed by the “Task” (red box) and a post-movement phase (blue box). Movement onset is indicated by the vertical black line.

Event-related desynchronization (ERD) or event-related synchronization (ERS) refers to a band power decrease or increase, respectively, relative to a reference period [88], [89], [90]. These phenomena are usually induced in response to a motor task and produce a reliable contralateral ERD and ipsilateral ERS in the alpha/mu (8–13 Hz) and/or beta (14–30 Hz) bands of scalp EEG. Thus, projecting the different ERD/ERS traces into a time-frequency plot for each electrode can further generate so-called ERD maps [91] to identify the spatial, temporal, and spectral characteristics of these signals. These features are based on a traditional neuro-structural model and have been robustly exploited using a simple task paradigm illustrated in Fig. 3 [92].

Fig. S1 provides an overview of the typical ERD/ERS patterns that are generated during various movement conditions for both able-bodied and participants with spinal cord injury (SCI). Here, we differentiate these conditions into: a) active, voluntary movement; b) passive movement and movement induced by functional electrical stimulation (FES); c) complex finger movement and peripheral stimulation; d) continuous finger, hand/arm movements; and e) motor imagery. We also indicate the same conditions for people with SCI, however, due to physical limitations of these individuals, there is no counterpart to c), and f) describes attempted movement instead of real movement.

The most classical ERD/ERS patterns appear in able-bodied participants when performing voluntary hand movements (Fig. S1a, h) [90], [93]. These contexts often produce a mu/beta ERD before and during movement, as well as a post-movement beta ERS (PMBS), also called the beta rebound. Active foot movement (Fig. S1a, f) can also induce a clear beta ERD followed by a PMBS [94], [95], [96]. These oscillatory activities are often also accompanied by an MRCP [97], [98].

During passive hand movements (Fig. S1b, h), there is no pre-movement ERD, however, a mu/beta ERD is present during hand movement (either completely passive, or facilitated by FES), followed by a PMBS [93]. A beta ERD and PMBS are also observed during passive dorsiflexion of both feet (Fig. S1b, f) [95]. A similar pattern occurs in the case of complex finger movement (Fig. S1c, h; cube manipulation) and additional median nerve stimulation. These tasks also induce a contralateral mu/beta ERD; where ipsilateral beta ERS returns quickly to baseline, the mu ERD/ERS oscillates [99]. These tasks also produce sensory evoked potentials that are much larger than the rest condition [100].

In addition to changes in oscillatory activity, movement trajectories (Fig. S1d, h) can be decoded from delta band activity [101]. For example, in [102], a mu ERD and characteristic delta band activity was observed when participants performed rhythmic finger movements (flexion, extension, repetitively). Along these lines, low-frequency time domain (LFTD) signals have been shown to encode hand/arm kinematic movement [103], [104], [105]. Importantly, for these and other works [106], there also exists a mu and beta ERD during movement as well as PMBS after the termination of movement.

Very early after the investigation of executed movements in EEG research, imagined movement or motor imagery (MI) (Fig. S1e, h) played a major role in BCI development. Mu and beta ERD occur during MI [90], [107], and are often followed by a PMBS [96], [108], [109] (Fig. S1e, f). These ERD/ERS signals are often focal phenomena that arise according to the topographic organization of the motor homunculus, similar to what was previously observed for motor execution tasks. For example, multimodal work using EEG source imaging and fMRI has revealed strong co-localization of sensorimotor cortex activation for hand and foot movement execution and imagery tasks [110], [111]. Similarly, common patterns of BOLD elevation observed in fMRI have also been observed using EEG source imaging [112] in the form of ERD in the sensorimotor cortex. Based on these findings, MI tasks involving body parts with spatially separated motor cortex representations often define the number of control signals that can be used. Many online BCI paradigms include four or more MI tasks of different body parts to control an end effector in multiple dimensions. Common tasks include imagining the action of the right hand, left hand, both hands, rest, foot, and tongue.

Studies have shown that the speed of hand movement or imagined hand movement is also correlated to EEG activity [113]. Many of these tasks can be easily detected and separated by a standard set of electrodes covering sensorimotor areas [114], [115], however, including subject-specific features from larger groups of electrodes can improve both offline and online performance [116], [117], [118]. Furthermore, successful discrimination of more specialized MI tasks of the same body part (i.e., hand gestures, limb joints) has shown promise in offline scenarios and may, in the future, be integrated into online control as well [119], [120], [121], [122].

In people with SCI, MRCPs are apparent for different movement attempts (Fig. S1f, h) as well as a beta ERD and, in some cases, a beta ERS [94], [123]. In a study on foot movement attempts, only a slight ERS was visible, which indicates that the PMBS is not solely triggered by afferent input but also by internal processes [95]. Passive foot movement in SCI patients (Fig. S1g, f) exhibits no visible patterns, which makes sense since there is no afferent feedback to the brain in these individuals [95]. In incomplete SCI patients, however, there may exist slight ERD/ERS activity.

Overall, when examining the existence of ERD/ERS patterns in people with SCI, several concerns need to be considered. First, attempting continuous hand/arm movement can lead to activity in sensorimotor and centro-parietal areas (hand-eye coordination). For example, in Pulferer et al. [124], delta band activity could be used for kinematic decoding in a SCI patient. Furthermore, a mu and beta ERD can also be observed in SCI patients performing hand and feet MI (Fig. S1i, f,h) [125], [126], [127], [128], [129], [130]. Finally, these patterns vary widely across patients and depend on the severity of their injury, the time since injury [94], [131] and also whether the individual had previous BCI training experience.

## IV. Capacity of Non-Invasive BCI

BCIs consist of three main pillars that facilitate the mutual learning of both the user and system with the intent to accomplish a task as easily as possible [132]. Considering and optimizing all three pillars - the user, the application, and the machine – is important to ensure the success of a BCI. While some labs and studies attempt to address all three of these pillars, focusing on a single one is much more common. Here, we will address some promising approaches to these concepts reported recently.

### A. The User–Training Strategies

**Fig. 4.**
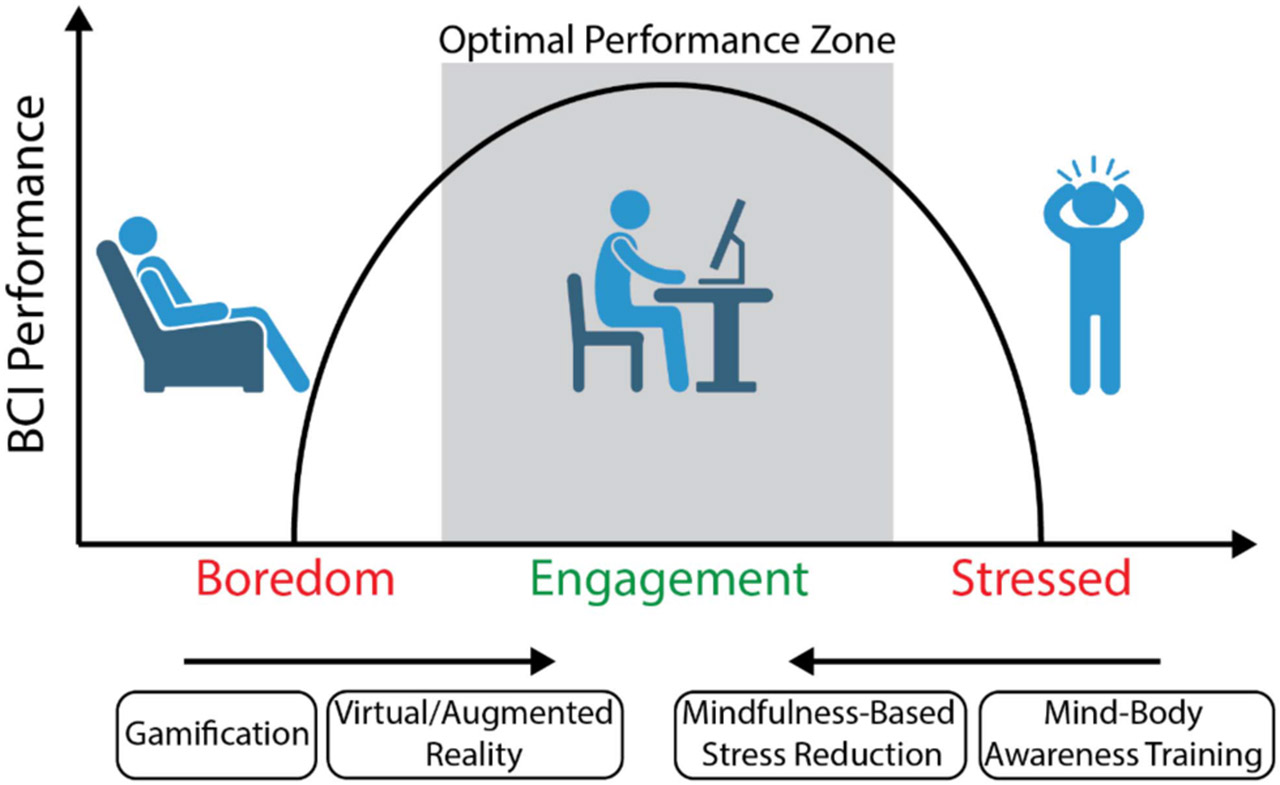
BCI strategies leverage principles of the Yerkes-Dodson law to improve performance. Virtual and augmented reality paradigms have been integrated into BCI control to increase user engagement. By contrast, various mediation-based practices help reduce anxiety levels. In both cases, the user’s mental state is driven towards an optimal performance zone where skill acquisition and execution can be maximized.

While some non-invasive BCI signals can be inherently produced, others must be learned throughout an often long training period. Some users never learn to successfully control a BCI despite lengthy training experience, which is often referred to as “BCI illiteracy” for a specific BCI paradigm. Thus, it is important to identify human factors that cause illiteracy and formulate strategies that promote BCI skill acquisition and optimal performance. One popular theory within the field posits that user attention plays a key role in the success of user training and achieving BCI control. This is often portrayed in terms of the Yerkes-Dodson law [133], which describes an inverted U-shape relationship between a user’s engagement and habit/skill formation. In this sense, tasks or contexts that are too simple can cause boredom, and those that are too difficult can cause stress, both of which negatively impact performance (Fig. 4). To address this, researchers have developed novel or adaptive tasks that appropriately engage subjects and improve BCI training and performance [134].

Along similar lines, many complementary technologies have been employed in BCI research to increase user engagement and avoid reduced performance due to boredom. Traditional BCI tasks that rely on endogenous signals utilize simple visual feedback paradigms involving a computer cursor, however, recent trends show an increased usage of virtual reality (VR) [135] and anthropomorphic feedback using computerized arms [10]. Previous cognitive studies have found that virtual hands and arms can elicit a strong sense of embodiment that is use-dependent [136], and suggest that these tools may also benefit BCI training. In fact, VR feedback with MI tasks has been commonly explored for motor recovery training in stroke patients with overall positive, but still varying levels of success [10], [14], [137]. These studies often focus on motor function rather than BCI performance and must accommodate highly variable brain damage, making it difficult to draw conclusions regarding the efficacy of VR aids.

Nevertheless, the use of VR tools with healthy individuals has shown much more promise by providing immersive contexts that enhance the embodiment of virtual limbs. Many of these studies have shown an increased amplitude of the respective ERD/ERS signals and a further improvement in online BCI performance compared to classic computer cursor feedback [138], [139], [140]. Using VR is currently a relatively low-cost addition to BCI systems; however, its full occupancy of the visual channel makes this technology somewhat impractical for future integration into daily life. By contrast, augmented reality (AR) is more compatible with everyday tasks and has been shown to be compatible with at least SSVEP-based BCIs [141], [142]. However, these contexts can create crowded visual scenes that must be considered so as not to overwhelm and distract the user [143].

At the other end of the spectrum, anxiety has been shown to impart a significant negative bias on BCI learning and performance for both MI and SSVEP-based BCIs [144], indicating that the self-regulation of mental state can lessen these effects. This factor has recently been creatively addressed through the use of meditation practice that can help alleviate elevated anxiety levels. Exemplary meditation-based strategies such as mind-body awareness training (MBAT) and mindfulness-based stress reduction (MBSR) training [145], [146], [147] have improved MI-based BCI performance. Overall, this suggests that acute and chronic stress levels impact BCI performance. Therefore, the ability to control a BCI may scale with the amount of stress-reduction training experience and the improved ability to produce stronger EEG signals associated with MI tasks.

### B. The Application–External Device Control

Modern brain-based navigation of external devices includes the control of computer cursors, quadcopters/drones, wheelchairs, and robotic arms. Computer cursor paradigms remain robust testbeds for exploring new decoding algorithms, tasks/signal types, and more. Currently, non-invasive cursor control can be performed in two [107] and three dimensions using MI tasks [116], [148], [149] or hybrid systems that utilize MI and OSA [66]. More sophisticated navigational control has also been achieved using virtual helicopters or drones [150], [151]. However, this application comes with additional complexity as continuous flight often requires an asynchronous control strategy with ongoing commands/tasks rather than discrete trials, such as the center-out paradigm. BCI-controlled continuous flight of a physical quadcopter in three-dimensional space was first demonstrated using ERD/ERS signals corresponding to MI tasks [152]. This feat was achieved using right- and left-hand MI for one dimension of control, and MI of both hands and rest for the second [152]. More recently, a combination of SSVEP and MI tasks was also used to control various aspects of quadcopter flight [153].

Another important application of BCI is the brain-based control of a wheelchair [154], [155]. Such a task allows BCI technology and the use of various brain signals, such as P300 or SSVEP responses, in addition to MI tasks, to assist impaired patients in situations resembling daily life [156], [157], [158]. A recent study demonstrated that tetraplegic patients with SCI can operate a self-paced wheelchair during complex navigation tasks using an MI protocol [155].

In recent years, multiple non-invasive BCI studies have focused on robotic arm control. Controlling robotics is far more complex than the previously described devices, as the increased physical skill required for grasping tasks requires a corresponding mental strategy. Initial demonstrations of robotic arm control via scalp EEG focused on endpoint velocity or position control. However, as more detailed control of reach-and-grasp tasks practical for daily assistance became desired, this work branched into additional threads that focus on individual task segments. These segments can be broadly categorized into navigational robotic device control (“reach”) and dexterous hand commands (“grasp”). Along these lines, the reaching aspect was initially demonstrated in three-dimensional movement decoding of center-out and motor execution tasks [159] and has since greatly expanded [160], [161], [162].

**Fig. 5.**
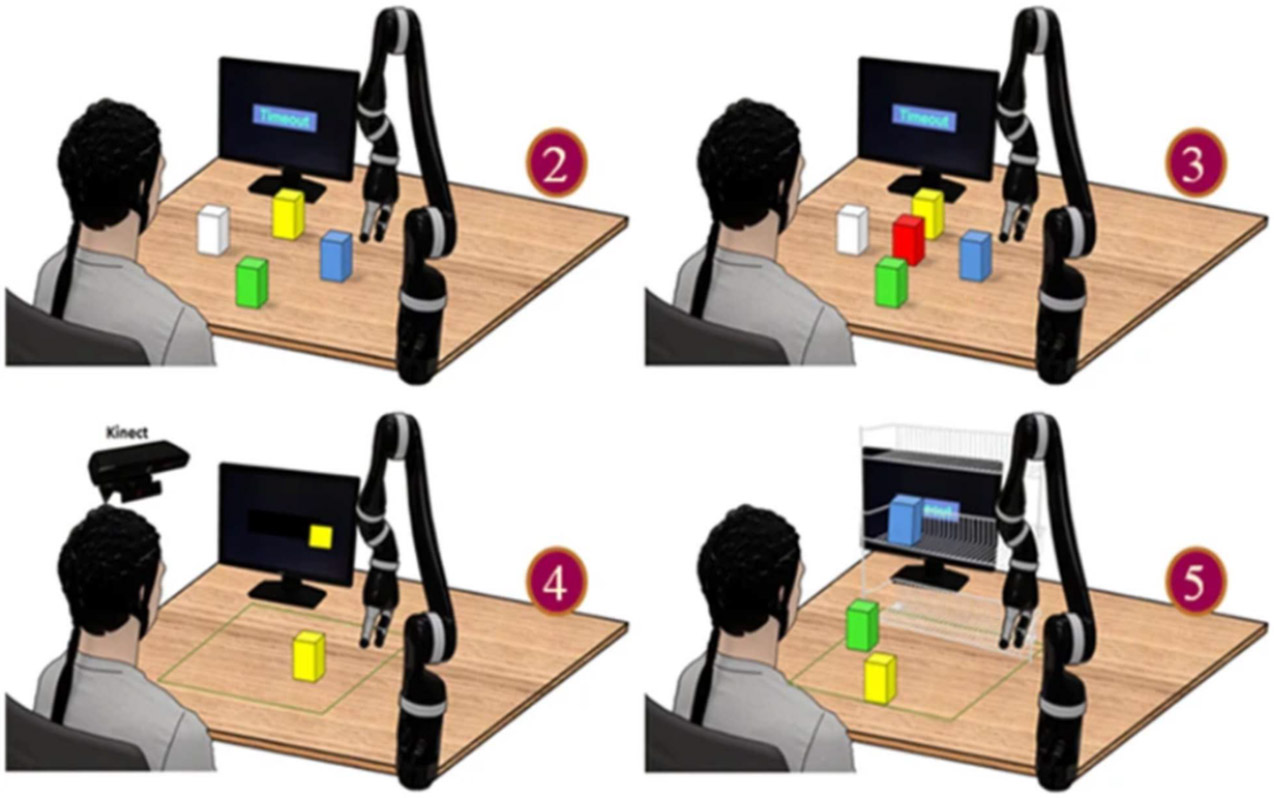
EEG-BCI based control of a robotic arm using motor imagery tasks. Motor imagery was performed to control the “reach” of a robotic arm in one and two dimensions. Similar motor imagery protocols were also used to perform the “grasping” of a lego block. Task complexity increases from 2 to 5 during subject training. Reproduced from Fig. 1 of [163].

Other than movement execution, MI paradigms have been pursued using ERD/ERS signals to control a robotic’s arm reach, grasp, and movement. He and colleagues have developed a unique and comprehensive framework for reach and grasp using MI. Along these lines, Meng et al. first demonstrated online non-invasive BCI control of a robotic arm in a group of human subjects, in whom MI tasks were used to reach for and grasp a Lego block (Fig. 5) [163]. Eight subjects completed all tasks and demonstrated well over 80% accuracy for completing reach tasks requiring two-dimensional navigation. Similar performance was achieved for more complex sequences of tasks in three-dimensional space involving grasping blocks off of a shelf, reaching over 70% accuracy. Edelman et al. further demonstrated the continuous reaching of a robotic arm through a combination of source-based decoding and a continuous pursuit control paradigm in a group of six human subjects [164], [165]. This work first demonstrated that the extended target-chasing task significantly improved MI-based BCI learning compared to traditional discrete trial protocols by increasing user engagement throughout training. By integrating real-time electrical source imaging, BCI performance was further enhanced across the skill spectrum in naive and experienced users. These works represent major advancements in the reach and grasp of BCI robotic arm control. When combined, this framework may, in the future, enable individuals with neuromuscular dysfunctions to autonomously perform daily tasks using only MI-based brain signals.

Robotic arm control has also been pursued from decoding movement execution. As such, to decode control initiation, Pereira et al. [166] developed a paradigm for online detection of self-initiated movements in a realistic scenario. EEG signals were time-locked to saccade onset, with participants shifting their gaze during goal-directed reach-and-grasp tasks. This strategy enabled relevant feature extraction and gaze incorporation with careful artifact handling [167]. A hierarchical classification approach utilizing LFTD features achieved a 54% true positive rate (compared to a chance level of 12%). This work expands possibilities for realistic settings, incorporating motor and visual processing in real-world tasks. Kobler et al. [103] further designed an experiment that involved center-out and continuous goal-directed movements, and considered two conditions (observation, execution) to study different volitional states. In either condition, the participants fixated on a target stimulus with their gaze in a 2D workspace. During motor execution trials, participants controlled a cursor by moving their right arm (on a flat surface) in a pursuit tracking task (PTT) to investigate the tuning characteristics of position and velocity. These temporal tuning characteristics indicated that neural activity preceded cursor velocity by approximately 150 ms [103]. These observations from EEG are in agreement with the tuning characteristics of spiking activity of M1 neurons [168].

In a follow-up study, Mondini et al. [105] investigated the feasibility of continuously decoding voluntary hand/arm movement trajectories from EEG to achieve closed-loop online control of a robotic arm. This work also implemented a PTT where the participants were asked to track a moving object on the screen by controlling a robotic arm. Kobler et al. [104] then suggested that integrating information about non-directional kinematics (e.g., distance, speed) in the decoding model can alleviate the amplitude mismatch problem. This non-directional kinematic information has been found in both ECoG [169] and MEG activity [170]. Provided that distance and speed are nonlinearly related to position and velocity, the previously introduced partial least squares Kalman filtering (PLSKF) approach [105] was extended to an unscented Kalman Filter (PLSUKF). Kobler et al. [104] re-analyzed EEG [103] and MEG data [170] to evaluate the performance of the PLSUKF with respect to both the PLS and the PLSKF during both observed and executed movements. Indeed, the correlation between the executed and the decoded trajectories was stronger for PLSUKF compared to other algorithms. Further, with the integration of non-directional kinematics in the decoding model, the amplitude mismatch between recorded and decoded trajectories could be reduced, overcoming the limitations of previous work [105]. Follow-up studies by Müller-Putz et al. and Pulferer et al. investigated the viability of decoding attempted movement, mimicking the limited motor function of SCI patients (by strapping each participant’s dominant arm to a chair) [86], [171]. They examined potential learning effects that may arise when a user performs the same motor control task multiple times [171]. Within five days, ten able-bodied participants performed three sessions. The selected timeframe aimed to allow participants to recover from the mental strain of various tasks while ensuring they maintained a distinct memory of previous sessions. After calibration, EEG decoding was gradually included in online feedback until it reached 100% EEG control. Overall, this longitudinal study revealed learning effects across the various days of training, indicating a positive effect of training on decoding performance.

While extensive effort has been dedicated to improving the navigation aspect of robotic arm control, considerable work has also focused on incorporating EEG-based grasping signals into a BCI. First attempts to restore hand movements in paralyzed persons were purely pragmatic in that BCIs were directly connected to an orthosis or the patients’ arm using FES [127], [128], [129]. These works demonstrated the possibility of connecting a BCI with closed-loop FES, recording EEG from the scalp, and stimulating a body part. However, they remained proof-of-concept studies. Study subjects were able to switch through pre-defined grasping patterns, but BCI control remained rather artificial in that they used the imagination of different limbs (foot and right hand) [129], or only the left hand [126] to trigger the different neuroprosthesis steps.

Nevertheless, in recognition of the various complexities involved with grasping objects of different sizes, shapes and orientations, further studies have since investigated how relevant grasping actions and features are encoded in scalp EEG. In particular, the use of MRCPs has helped support the hypothesis of natural control of a neuroprosthetic device. MRCPs vary across different types of movements or grasps, which may be useful as a natural control signal for a BCI-based neuroprosthetic. In fact, studies with able-bodied participants have shown that different movement types (right arm: hand open, hand close, pronation, supination, elbow flexion, elbow extension), grasp actions (i.e., palmar, pincer, lateral, rotation), grasp laterality (i.e., unimanual vs bimanual) and grasp force (i.e., power vs precision) can be successfully decoded from scalp EEG using MRCPs [172], [173], [174], [175], [176], [177], [178], [179]. Importantly, similar differences across multiple grasp types at the single-trial level could also be observed in individuals with SCI [123].

While these studies show promise for neurorobotic control, they were primarily employed in offline classification settings. Translating these offline studies into online performance greatly increases the potential utility in patient-oriented BCIs. Working towards this goal, some studies that integrate decoding paradigms that simulate online control, have shown successful above-chance performance for the execution of three different grasp types (i.e., palmar, lateral, wrist supination) [180]. More recent work has successfully moved to complete online control of four grasp types (i.e., cylindrical, spherical, lateral, pincer) using MI tasks [181].

This demonstration indicates that dexterous grasping can be performed in real-time using scalp EEG, however, it is difficult to suppress reaching signals during the grasp portion of the task, and vice versa. In this case, mode switching would be greatly beneficial in informing the BCI whether the user is focusing on positioning the arm or grasping an object. Similar to the previously described movement onset detection, various BCI “switches” have been proposed, but have also yet to be seamlessly integrated into online control paradigms [182]. Therefore, an easier solution to robotic arm control for daily tasks that has been widely adopted by the field is to utilize shared control strategies [183], [184], [185], [186]. In this sense, shared control refers to partial autonomous control by the user and partial automatic task recognition and execution by the BCI [187]. In these cases, such a strategy allows the user to control the BCI in up to three dimensions using a variety of MI and SSVEP signals (often hybrid) to select a pre-established task/action that is then automatically carried out. Nevertheless, the degree and complexity of shared control vary. Some systems utilize custom image processing pipelines for object identification using off-the-shelf camera components such as the now-discontinued Xbox Kinect. However, this approach can be limited to objects with similar shapes consistently viewed in the same orientation [184], [185], [188]. Other strategies utilize depth cameras and more advanced pose estimation methods [189] or simple QR codes [186] to identify objects that may be approached from different angles or perspectives, potentially offering a more versatile solution.

To ensure effectiveness and safety, the user and machine must consider practical considerations when utilizing a real-life robotic arm. Recent work on robotic arm control utilizes SSVEP signals for at least one control dimension. However, the visual attention required for these signals can be distracting when integrating these systems into everyday life. Similar issues have been observed when controlling a robotic arm using MI tasks – while the visual channel is reserved for observing the robotic arm rather than the flickering stimuli, impaired sightlines can still cause a slight drop in performance [165]. It has also been shown that other cognitive distractions accompany the user when controlling an interactive device, which can reduce its effectiveness [190], [191]. Another practical consideration for robotic device control that has been recently addressed is the need to impose physical constraints such as obstacle avoidance or trajectory correction [192], [193] to ensure the user’s and others’ safety. Controlling a robotic arm is a highly complex cognitive process, and various precautions and aids should be added to BCIs to maximize their effectiveness.

### C. The Application–Clinical Use

Non-invasive BCIs have emerged as a transformative technology with significant clinical applications, and carry immense potential for improving the quality of life for individuals with various neurological conditions and disabilities. Using BCI technology for clinical applications can complement traditional strategies for disease diagnosis, treatment, and rehabilitation. For example, BCIs facilitate the early detection of EEG biomarkers for epilepsy monitoring and intervention. In addition, BCIs interact directly with the brain, enabling precise targeting of neural pathways. This can lead to more accurate and effective treatments of specific bodily functions and provide immediate feedback based on brain activity. Finally, the interactive nature of BCIs can be more engaging and motivating for patients than repetitive physical exercises.

BCI technology has two primary clinical applications: 1) assistive BCI and 2) rehabilitative BCI [194]. Assistive BCI seeks to bypass damaged neural pathways, providing a continuous and permanent means of communication and control of external devices. Assistive BCI systems are designed for individuals with severe motor impairments. These systems use SSVEP, P300, or MI signals to control various external devices, such as spellers [195], computer cursors [196], robotic devices [197], or speech synthesizers [198], thereby improving the user’s quality of life and independence. Rehabilitative BCI aims to recover damaged neural connections, restoring impaired functional abilities by effectively facilitating neuroplasticity. This approach offers comprehensive and personalized functional, cognitive, and affective rehabilitation therapies. Restoring motor function is one of the most promising clinical applications of BCIs for individuals with SCI, stroke, or neurodegenerative diseases such as ALS. Since conditions like stroke do not affect the ability to form motor intentions, BCIs can decode neural signals related to these activities. This allows for neurofeedback training through assistive devices such as robotic systems, virtual reality, and functional electrical stimulation. Despite the significant attention BCI-based rehabilitation has received for motor function, applications in cognitive training remain underexplored. Evidence suggests that BCI-based neurofeedback training can improve cognitive functions in conditions like attention-related hyperactivity disorder [199] and mild cognitive impairment [200], and may similarly benefit post-stroke cognitive impairments [201]. These findings highlight the potential of neurofeedback-based cognitive training to induce neuroplastic changes essential for rehabilitation. BCI-based interventions have also demonstrated significant potential in emotion and mood regulation, offering solutions to address critical challenges such as depression. By leveraging real-time neural feedback, these interventions enable individuals to consciously modulate their brain activity and promote more positive emotional states. Advanced BCI systems can detect and respond to specific neural patterns associated with mood disorders, providing personalized therapeutic feedback that encourages neural reorganization and emotional resilience. Such interventions not only complement traditional treatments but also offer a non-invasive, self-regulated approach to managing and mitigating depressive symptoms, thus opening new avenues for mental health care. We believe the future of BCI for clinical applications lies in integrating functional, cognitive, and affective rehabilitation into a holistic approach. This comprehensive strategy enhances motor recovery and supports cognitive and emotional health, paving the way for more robust and inclusive rehabilitation solutions.

Compared to BCIs targeted at healthy users, BCIs designed for clinical applications still present several unique challenges. Patients usually show higher individual variability in neurological conditions. Patients may also experience higher levels of fatigue and cognitive load when using BCI systems. Furthermore, neurological impairments can affect the quality of neural signals that the BCI relies on. Many neurorehabilitation patients may have limited mobility or dexterity, making it difficult for them to use BCI hardware comfortably. Consequently, recent advancements propose the use of advanced decoding methods [202], [203], [204], [205] and the integration of other medical interventions, such as transcranial direct current stimulation (tDCS), transcranial magnetic stimulation (TMS), and transcranial focused ultrasound stimulation (tFUS) [10], [26], [27], [206], [207], [208] to develop more robust, calibration-free, and user-friendly BCIs for clinical applications.

### D. The Machine–BCI Decoding Algorithms

EEG decoding involves developing algorithms and models to analyze recorded EEG signals, with the aim of understanding and interpreting the underlying neural processes. EEG decoding is crucial for BCIs because it enables the translation of neural signals into actionable commands, realizing communication and control between the human brain and external devices. Brain signal decoding is always a challenging part of BCI, especially for non-invasive EEG. As EEG signals travel from their source in the brain to the scalp surface through the low-conductive skull, they encounter various instances of noise caused by both biological and non-biological factors.

While EEG signal decoding can be viewed as a specific type of classification problem, this topic presents unique complexities. In typical classification tasks, larger datasets enhance performance by providing richer information. However, in EEG classification, more data necessitates longer acquisition times, which can lead to participant fatigue and distraction. This degrades data quality, introduces noise and inconsistencies, and negatively affects model training. Therefore, proper EEG classification needs a balance between data quantity and quality. It is generally recommended to limit EEG recording sessions to 30–60 minutes, with regular breaks, to maintain high data quality. For intra-subject training, a model is trained and tested on data from the same subject. By contrast, inter-subject training involves training a model on data from multiple subjects and testing it on different subjects. This requires a large and diverse dataset to capture the variability in EEG signals across individuals, along with more complex operations to create a model capable of handling EEG data with different distributions.

The traditional classification pipeline in BCI typically encompasses an initial filtering process applied to EEG signals, followed by the classification of the filtered data. This filtering operation is designed to extract pertinent information from temporal, spectral, or spatial dimensions, depending on the specific BCI paradigms. However, traditional methods face many challenges when decoding EEG. First, the feature extraction and classification processes in traditional methods are optimized separately using distinct objective functions. This could result in a suboptimal solution for the decoding process. Second, traditional methods strongly rely on hand-crafted features, a manual selection process that can be time-consuming and subjective. Finally, while these approaches prove effective in a specific subject, their performance tends to decline when applied in subject-independent applications. Therefore, more advanced methods are explored to address these limitations.

**Fig. 6.**
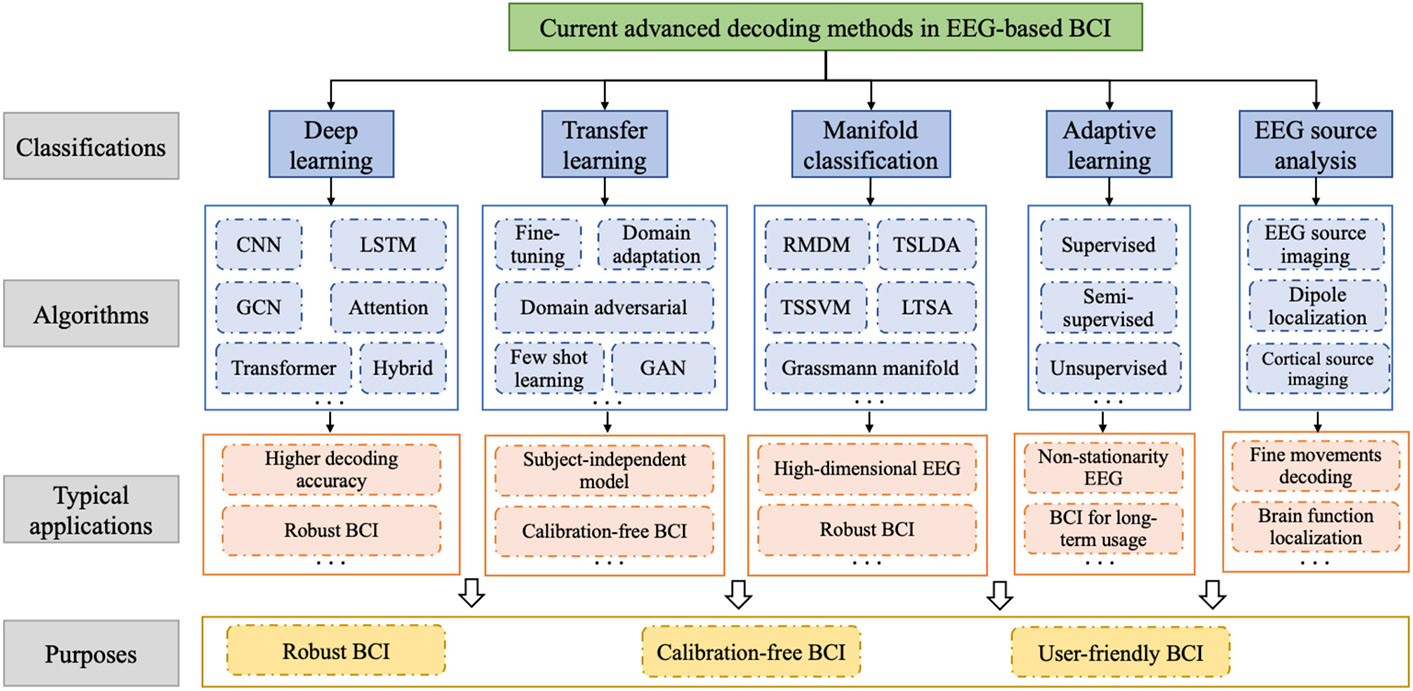
Overview of recent decoding methods used in EEG-based BCI. These methods can be broadly separated into five main categories: Deep learning, transfer learning, manifold classificiation, adaptive learning, and EEG source analysis. Each of these categories consists of detailed algorithms that aim to improve various aspects of EEG decoding and BCI performance. In the end, all of these approaches aim to develop robust, calibration-free and user-friendly BCIs.

In this section, we comprehensively explore recent progress in decoding methods for EEG-based BCI. We outline the principles underlying these methods, how these methods relate to one another, and provide guidance on their appropriate application. The methods discussed in this section are summarized in Fig. 6 and Table I.

#### 1) Deep Learning-Based Methods:

Deep learning is a subfield of machine learning that focuses on the development and application of artificial neural networks to solve complex problems such as computer vision, speech recognition, and natural language processing [209]. With their ability to automatically learn hierarchical representations from raw data, deep learning approaches offer a promising alternative for EEG decoding and have achieved state-of-the-art performance.

A convolution neural network (CNN) is among the most popular and successful deep learning methods for EEG decoding. CNNs are designed for signal processing and analysis, especially for those contexts that provide spatial information. The convolutional layer is a fundamental building block of CNN that extracts features by sliding small filters across the input. Each filter is equipped with learnable weights designed to detect specific patterns, and the aggregation of multiple filters results in the creation of a feature map. During the training of a CNN, backpropagation is adopted to calculate the gradients of the loss function with respect to the weights of the network, allowing for weight updates that minimize the overall loss. When adopted into EEG decoding, CNNs are generally initiated with an input layer that accepts the pre-processed EEG data. Then, the CNN extracts spectral and spatial features by deploying a spatial convolution layer along the channel dimension and a temporal convolution layer along the sample dimension. After this step, several layers will be followed to project the feature into higher dimensions. Between two convolutional layers, there always exists one pooling layer to reduce the parameter dimension and eliminate overfitting. In the final phase, the fully connected layer serves as a decision-making layer, mapping the high-level representations to the specific classes related to the decoding task. Many current approaches are completely or partially based on a CNN. In a typical and pioneering work, Cecotti & Graser [210] proposed a CNN-based method for P300 decoding. This network was quite simple yet effective, with only two convolution layers and one fully connected layer. As one of the initial papers to apply the CNN to BCI, Sakhavi et al. [211] designed a framework to learn temporal information from EEG, and achieved the best classification performance on BCI competition IV-2a dataset. Subsequent efforts aim to enhance performance by incorporating structures with varying convolution depths and by employing advanced training strategies. Two CNN-based classifiers named Shallow ConvNets and Deep ConvNets were introduced for MI decoding in Schirrmeister et al. [212]. The Shallow ConvNets were initiated with a spatial convolution layer and a temporal convolution layer to extract features from the spatial and spectral domains, respectively. Deep ConvNets was equipped with three more convolution layers that project outputs into higher dimensions, facilitating the learning of features in more complex spaces. Results showed that Deep ConvNets reached at least as good performance as filter bank common spatial patterns (FBCSP), a staple of the field [213]. The EEGNet proposed by Lawhern et al. [214] demonstrated a high level of versatility of the CNN-based method on four different BCI paradigms, i.e., P300, error-related negativity (ERN), MRCP, and ERD/ERS. This method introduced depthwise and separable convolutions for feature extraction and achieved high classification accuracy while using fewer parameters than other models. In more recent work, Mane et al. [215] proposed a filter-bank convolutional network (FBCNet) to classify MI tasks from EEG for both healthy and stroke subjects. FBCNet employed a multi-view data representation followed by spatial filtering to extract spectro-spatially discriminative features. A similar work introducing multi-scale CNN to learn spatial-temporal features for epileptic seizure detection can be found in [216].

EEG signals are collected by multiple electrodes positioned on the scalp, inherently forming a spatial network. To consider the physical distribution of EEG signals, graph convolutional networks (GCNs) were introduced as an extension of CNN. In a graph, data are represented by nodes and edges, and convolution involves aggregating information from neighboring nodes. The concept of a “neighborhood” holds different meanings for traditional convolution and graph convolution. For traditional convolution, a neighborhood refers to local patches or regions of the input data. For graph convolution, however, it refers to the set of adjacent nodes connected by edges. When decoding EEG using a GCN, the data are first represented as a graph with nodes (electrodes) and edges (relationship between electrodes). This can be realized by calculating the absolute Pearson’s matrix of EEG data [217]. Then, the initial feature vectors are assigned to each node in this graph. Finally, graph convolutional layers are implemented to capture relationships between connected nodes. GCNs have been used for MI classification [218], P300 decoding [219], and cognitive analysis [220] by learning the intrinsic relationship between different EEG channels. Recently, a CNN variation that considers the “neighborhood” of EEG electrodes – PointNet – has been introduced to study continuous tracking in MI BCI [221].

A recurrent neural network (RNN) assumes that there are long-term dependencies in sequential data. RNNs capture information from previous time steps by maintaining hidden states [222], are capable of decoding temporal and spectral information from EEG. Long short-term memory (LSTM) is a typical RNN, which was proposed to address the vanishing gradient problem in RNN by incorporating a memory cell and three gates named forget gate, input gate, and output gate. The memory cell is capable of maintaining long-term memory, while the three gates control the flow of information into and out of the memory cell. This structure enables LSTM to handle sequences of varying lengths and retain important information over extended periods. Totora et al. [223] proposed an LSTM network with two separate layers, allowing the network to capture both low-level and high-level gait patterns from brain signals.

Despite the effectiveness of CNNs and LSTM in capturing spectral and temporal features in sequential EEG, they encounter challenges when dealing with long-range dependencies and may experience a long training process to selectively focus on crucial elements in EEG. To address this limitation, attention mechanisms were introduced [224]. In EEG decoding, the relevant information for making a prediction is not equally distributed across the entire trial. Attention mechanisms enable the model to dynamically weigh different parts of the input, giving more importance to relevant regions. In the attention mechanism, the “query” represents the current position or context that the model is focusing on and the “key” represents different positions or elements in the input data. Attention is realized by calculating attention scores, which represent the importance of each key with respect to the current query. The attention mechanism has been used in ERP classification [225] and VEP detection [226]. One typical attention-based method is the transformer, which uses multi-head attention. This involves multiple parallel selfattention mechanisms, allowing each position in the sequence to attend to all positions. Transformers have been used for MI decoding [227], ERP detection [228], SSVEP recognition [229], etc.

The previously presented deep learning methodologies are characterized by singular architectural frameworks. Hybrid deep learning (hDL) methods, on the other hand, combine the diverse strengths of different decoding approaches to effectively integrate temporal, spectral, and spatial features from EEG data, thereby offering a comprehensive solution for EEG decoding. The most common hDL approaches combine homogenous CNNs together, or integrate CNNs with other neural networks, including RNNs, transformers, and other variants. [230] combined a CNN with a multilayer perceptron (MLP) to facilitate epileptic seizure prediction. A CNN-LSTM framework was proposed to extract the spectral, spatial, and temporal features from EEG to address the BCI illiteracy problem in MI paradigms [231] and also to boost the classification of SSVEP paradigms [232]. Song et al. [233] introduced a compact convolutional transformer to enhance the decoding of SSVEPs by encapsulating local and global features in a unified framework. Despite hDL’s potential for a more comprehensive analysis of EEG signals, its elevated model complexity and computational costs still pose challenges in BCI.

The appeal of deep learning methods for EEG decoding lies in three main reasons. First, the EEG signal is inherent with high variability across time and subjects, leading traditional methods to fail to generalize well across these diversities. On the contrary, deep learning methods are capable of integrating high-level knowledge directly from raw EEG, significantly reducing the reliance of the decoding algorithm on prior knowledge. Second, deep learning methods are trained in an end-to-end manner rather than the two-step phases employed in traditional methods, ensuring a global optimum during the decoding process. Finally, deep learning methods automatically extract features, avoiding the highly time-consuming and labor-intensive extractor designing process of traditional methods. However, it is important to note that deep learning methods assume the training and testing data share the same distribution, an assumption that may not hold in many real-world scenarios. Furthermore, the challenge of obtaining extensive and diverse labeled training data for training a deep learning model is often not feasible in practical cases like clinical applications. Therefore, despite their advantages, deep learning methods still face challenges, and more advanced methods should be explored.

#### 2) Transfer Learning-Based Methods:

Transfer learning is currently a hot topic in machine learning and data mining. Different from deep learning methods, transfer learning assumes that the distribution of training and testing data is different, and tries to utilize knowledge learned in one group to solve problems in another [234]. In transfer learning, the domain comprises the feature space 𝒳 and the probability distribution P(X), where $X=\{x_{1},x_{2},\ldots ,x_{n}\}\in 𝒳$, with n being the number of feature vectors. In a specific EEG decoding problem, X is all available EEG feature vectors in this decoding task with $x_{i}$ denoting one particular vector extracted from one single EEG trial. n can therefore also be considered as the number of EEG trials, and 𝒳 the feature space of all EEG samples. In transfer learning, the domain holding known knowledge is termed the source domain, typically denoted as ${𝒟}_{𝒮}$. The domain encompassing unknown knowledge to be acquired is termed the target domain, usually represented as ${𝒟}_{𝒯}$. The learning goal is referred to as a task 𝒯, which consists of a label space 𝒴 and the prediction function f(⋅), and $Y=\{y_{1},y_{2},\ldots ,y_{n}\}\in 𝒴$ is the label space in that training dataset. More formally, given a source domain ${𝒟}_{𝒮}$ with task ${𝒯}_{𝒮}$ and a target domain ${𝒟}_{𝒯}$ with task ${𝒯}_{𝒯}\;({𝒟}_{𝒮}\neq {𝒟}_{𝒯})$, transfer learning aims to utilize the information learned from ${𝒟}_{𝒮}$ to facilitate the prediction function $f_{T}(\cdot )$ [235]. According to the label type of available data, transfer learning can be divided into inductive transfer learning, transductive transfer learning, and unsupervised transfer learning. In inductive transfer learning, the labels of the target domain are known, and the target task and the source task are different (${𝒟}_{𝒮}\neq {𝒟}_{𝒯}$). In unsupervised transfer learning, data from both the source and target domains are unlabeled, and tasks in the two domains are different (${𝒟}_{𝒮}\neq {𝒟}_{𝒯}$). Most BCI research focuses on transductive transfer learning, where ${𝒟}_{𝒮}\neq {𝒟}_{𝒯}$, but ${𝒯}_{𝒮}={𝒯}_{𝒯}$. For instance, in the context of a group of subjects performing an identical MI task, the labeled EEG data from some subjects (source domain, ${𝒟}_{𝒮}$) are used to improve the decoding of the unlabeled EEG signals in naive subjects (target domain, ${𝒟}_{𝒯}$).

To date, the predominant approach in transfer learning for BCI has largely relied on fine-tuning techniques. In one typical cross-subject fine-tuning application, one subject-independent model $f_{S}(\cdot )$ will be pre-trained on all of the data from ${𝒟}_{𝒮}$, and then the data from ${𝒟}_{𝒯}$ will be used to fine-tune a subject-dependent model $f_{T}(\cdot )$ on $f_{S}(\cdot )$ [236]. Zhang et al. [237] demonstrated the efficacy of fine-tuning using a pre-trained model from other subjects in cross-subject applications. Besides this, fine-tuning method can also be used in cross-session scenarios, where the data from the calibration session are pre-trained to initialize a model, while data from the subsequent sessions are adopted to fine-tune a session-specific model [238]. Fine-tuning has been widely used in MI decoding [239], SSVEP detection [240], and P300 recognition [241]. Previous studies with deep learning have shown that fixing some of the initial weights in $f_{S}(\cdot )$, while adjusting others by the optimizer, will improve the performance of transfer learning. This technique is termed frozen and has been adopted into BCI to enhance the decoding performance of the fine-tuning strategy [242]. Notably, not all subjects from the target group will benefit from the performance of the source group. Coarsely performing a transfer regardless of the dissimilarity between the source and target domain will lead to even worse decoding accuracy. This phenomenon is called “negative transfer” [243]. To tackle this issue, domain adaptation techniques are proposed in EEG decoding.

Domain adaptation techniques are employed to adapt the source domain information to better align with the features of the target domain. This approach was introduced in EEG decoding to help mitigate the variability between different subjects, sessions, or experimental conditions. Domain adaptation can be realized by conditional distribution adaptation and marginal distribution adaptation, i.e., with or without considering the relationship between variables. Conditional adaptation considers the joint distribution of multiple variables, seeking to preserve the relationships that exist in the data. It not only aligns individual feature distributions but also aims to capture the interdependencies and relationships between features. On the other hand, marginal distribution adaptation performs domain adaption without considering the relationship between variables. Chen et al. [244] proposed a multi-source marginal distribution adaption method. First, a common feature extractor was designed to map the sources and target data from the original feature space into a common shared latent space. Then the EEG from the target domain and source domain was combined as pairwise inputs to learn domain-specific features. In order to project the data from the target and source domain as closely as possible, they adopted a maximum mean discrepancy (MMD) loss to obtain domain-invariant features. So far, more and more work on domain adaption concern both marginal distribution and conditional distribution to adapt models to new subjects or datasets while preserving task-specific information [245], [246].

Domain adversarial is a relatively new approach to realize domain adaptation, aiming to learn a model that cannot identify the domain of origin of the input observation. This idea is based on the belief that if an algorithm cannot distinguish the source domain based solely on the transformed representation, it indicates that the representation has effectively abstracted domain-specific information. When performing domain adversarial, data with both class labels and domain labels are initially input into a feature extractor. These representations are then directed towards a label predictor and a domain classifier. The objective is to minimize loss in the label predictor while employing a gradient reversal layer to maximize loss in the domain classifier. This process reduces domain-specific information within the representation space and significantly improves the generalization ability of the label predictor. Inspired by this idea, Li et al. [247] trained subject-independent models by reducing the impact of subject-specific information on the EEG emotion recognition process. Liu et al. [248] proposed a unified multi-source optimization framework to realize multi-source domain adaption by learning the weight distribution and fusion results.

Besides realizing domain adaptation, adversarial training is also adopted to perform data augmentation by generating artificial samples resembling the real data distribution it was trained on. A generative adversarial network (GAN) is another transfer learning method. It consists of a generator to create EEG data and a discriminator to distinguish between the real and artificially generated samples. The training process of a GAN involves a continual back-and-forth struggle between the generator and the discriminator. It succeeds when the generator can produce synthetic data that are indistinguishable from the real data by the discriminator. Typically, this method can be used for EEG data augmentation [249], [250] and artifact detection [251]. Training a GAN using EEG is widely acknowledged as a tough task due to prevalent instability issues. One notable challenge arises when the discriminator quickly reaches an optimal state, effortlessly differentiating between the synthetic samples and the real EEG samples. As a consequence, this will lead to no meaningful gradients and bring no progress for model training.

While improving classification accuracy, transfer learning is also capable of achieving a calibration-free BCI. Few-shot learning is an effective transfer learning tool for achieving this goal [252]. This approach trains models to make accurate predictions or classifications with very few labeled examples, or even without any supervised information (zero-shot learning). There exist three primary approaches to facilitate few-shot learning. The first option is to augment the training dataset. Besides the above-mentioned GAN, data augmentation can be realized by incorporating various representations with temporal EEG trials (such as spatio-temporal features and power spectral density) [253], by designing a sliding window to crop EEG data into smaller segments across the temporal domain [212], by injecting various types of noise (such as Gaussian, Poisson, etc.) into clean EEG to create new EEG trials [254], and by performing oversampling [255] or subsampling [256] on EEG trials. The second option is to constrain the hypothesis space within the model. An et al. [257] proposed a few-shot learning method by using two branches of embedding modules to extract data from support data and query data. This work was applicable to few-shot learning because, instead of learning features within each EEG trial individually, it focused on learning the relationships between EEG trials, which significantly constrained the hypothesis space. The third option is to modify the search strategy within the hypothesis space. The previously mentioned fine-tuning approach serves as a common search strategy, refining existing parameters from source tasks to optimize the parameter for the target task.

BCI datasets typically have limited samples due to the challenges in collecting brain data, making transfer learning an ideal solution for signal decoding. Transfer learning has shown its priority in training subject-independent models. It is worth noting that prior to executing the transfer process, careful consideration of domain adaptation is recommended to prevent negative transfer.

#### 3) Manifold-Based Methods:

Manifold approaches are different from the classification algorithms we have discussed until now. The feature space of these algorithms is defined in Euclidean space, which is flat everywhere. Manifold space, on the other hand, exhibits flatness at a specific point and its vicinity [258]. The manifold theory assumes that the higher-dimensional EEG feature space is mapped from a lower-dimensional space. Throughout this mapping procedure, the feature space introduces dimensionality redundancy and interferes with the feature extraction process. Consequently, manifold-based classification aims to unveil the inherent structure or geometry of this high-dimensional data, represent it in a more concise lower-dimensional space, and then perform classification.

Among different manifold-based classification methods, the Riemannian manifold is the most commonly used for EEG decoding [259]. This method aims to pull symmetric positive definite (SPD) features of the same class toward their barycenters and separate other features of different classes farther away in Riemannian space [260]. SPD features are important for calculating the Riemann distance because its symmetry ensures the Riemann distance remains invariant under coordinate transformations and its positive definiteness guarantees the well-defined nature of the Riemannian metric, ultimately resulting in a meaningful distance measure. In EEG decoding, the SPD matrix is typically derived from the covariance matrix of EEG trials, represented as $C=\frac{1}{N-1}XX^{T}\in R^{N\times N}$, where N is the number of EEG channels. This SPD matrix characterizes the relationships among various electrodes and serves as an effective tool for capturing the characteristics of EEG signals. Given the two SPD matrices $C_{1}$ and $C_{2}$, the affine-invariant Riemann distance δ between them can be calculated by [317], [318]:

(1) $$\delta_{R}\;(C_{1},C_{2})={\left\|\log\left(C_{1}^{-1∕2}C_{2}C_{1}^{-1∕2}\right)\right\|}_{F}={\left[\sum_{i=1}^{N}\frac{1}{2}\log^{2}\lambda_{i}\right]}^{1∕2}$$

where $\lambda_{i},i\;\;=\;\;1\ldots N$ are the real eigenvalues of $C_{1}^{-1∕2}C_{2}C_{1}^{-1∕2}$. By using the Riemann distance, the centroid of I SPD matrices on a Riemannian manifold (also known as the Riemannian geometric mean point) can be obtained through the following formula:

(2) $$G\;(C_{1},\ldots ,C_{I})=\underset{C\in C(n)}{\text{argmin}}\sum_{i=1}^{I}\delta_{R}^{2}(C,C_{i})$$

where C(n) is the space of the SPD matrices and C is the SPD matrix representing one point in the manifold. When decoding EEG in a Riemannian manifold, all SPD matrices of the EEG trials are first mapped into the Riemannian manifold space. Subsequently, the Riemannian geometric mean points of every class are computed. When new test data are introduced, the Riemann distances between this point and the Riemannian geometric mean points of each class are calculated. The point is then classified into the class associated with the minimum Riemann distance. This method is called Riemannian Minimum Distance to Means (RMDM) [319], and is parameter-free, robust to noise, and therefore exhibits better generalization capability. Other solutions try to introduce traditional classifiers like linear discriminant analysis (LDA), support vector machine (SVM), and neural networks into classification. As these methods rely on Euclidean geometry assumptions (LDA and SVM assume the decision boundaries are linear in the input space, and neural networks heavily rely on optimization using gradient descent based on Euclidean geometry), additional operation projecting data points from Riemannian space into tangent space is employed. The flat and linear nature of the tangent space at a specific point serves as an approximation of Euclidean geometry at that point and thereby meets the assumption of traditional classifiers. This concept has led to the development of Tangent Space LDA (TSLDA) and Tangent Space SVM (TSSVM) for EEG decoding.

Manifolds based on Riemannian geometry have been successfully adopted in EEG decoding. A novel geometric deep learning-based model was proposed in [261] to learn spatiotemporal representations of EEG data fully on a Riemannian SPD manifold. This work was tested on MI, ERP, and SSVEP datasets. Apart from the Riemannian manifold, many other manifolds have been explored in recent years. Gunawardena et al. [262] proposed a kernel-based non-linear manifold classifier to identify important channel inter-relationships within the EEG data. Li et al. [263] extended the low-rank representation method from the Euclidean space to the Grassmann manifold, aiming to investigate subspace information and re-represent EEG signals.

Manifold-based classification can be performed in an unsupervised manner, allowing the decoding of EEG data without the need for labeled examples. Moreover, the robustness of manifold-based methods extends to their ability to handle noise and outliers in the EEG signals, making them more applicable to real-world scenarios.

#### 4) Adaptive Learning-Based Methods:

An adaptive classifier is a type of classification algorithm that can adjust its parameters or structure based on the characteristics of the data it is processing. The adaptability of such a classifier allows it to respond to changes in the input data distribution, making it more flexible and potentially better suited for dynamic or evolving environments. Adaptive classifiers can be categorized into three types: supervised adaptation, unsupervised adaptation, and semi-supervised adaptation. For supervised adaptation, all data used for adaptation are supposed to be labeled. Most of the adaptive methods for EEG decoding are based on supervised adaptation. One typical application is cross-session adaptation for the long-term utilization of BCI, which requires the method to continuously adapt to the subjects’ mental state with the incoming unlabeled data. In this case, the decoding algorithms aim to retrain a new model to classify the unlabelled data from session i with the data from session 1 to i−1 in a supervised manner [238].

On the contrary, for unsupervised adaptation, no labeled data are available in the target domain. In such cases, the model has to rely on domain information or other unsupervised learning techniques to adapt itself to the new domain. Most works using unsupervised adaptation for EEG decoding attempt to learn domain-invariant features by training a domain-adversarial or domain-alignment model [264], [265].

For semi-supervised adaptation, most of the EEG used for model training is unlabeled, while only a fraction of them is labeled. Given that data labeling can be time-consuming and tedious, semi-supervised adaptation may have more potential than supervised adaption in EEG decoding. Pseudo-labeling, self-training, and co-training are three semi-supervised methods used for EEG decoding. In pseudo-labeling, the initial EEG decoding model is trained on the labeled EEG dataset. Then, the trained model is applied to the unlabeled EEG data to generate pseudo-labels. Finally, the labeled and pseudo-labeled datasets are combined together to retrain the model. This process is usually iterated many times to refine the model and update the pseudo-labels [266]. Self-training is similar to pseudo-labeling, except only those predictions with high confidence are added to the labeled set for the next iteration of training [267]. In co-training, multiple models are trained on different subsets of EEG features. The models then vote on the labels for the unlabeled EEG, and the most confident predictions are added to the labeled set [268]. It has been proven that when using the proper method, the semi-supervised method can reach a similar performance as the supervised method [269].

Compared with static methods, adaptive learning-based methods show more advantages in the real-time adjustment and robustness to non-stationarity for EEG decoding. Therefore, they are particularly well-suited for real-world applications.

#### 5) EEG Source Analysis:

The adoption of source domain analysis for EEG decoding introduces a departure from the conventions observed in sensor domain analysis that have been previously discussed. Sensor domain analysis deals directly with the signals recorded by EEG sensors on the scalp, focusing on patterns and features at the sensor level. On the other hand, source domain analysis seeks to go beyond the scalp-level signals to estimate the neural sources responsible for those signals, providing more detailed spatial information about brain activity. He and his colleagues have pursued source domain analysis [270], [271], [272] for BCI for years and have achieved equal or superior performance compared to sensor domain analysis [121]. Early work on this topic investigated the performance of using EEG source imaging using cortical current density or equivalent dipole models to classify MI tasks without BCI feedback [121], [273], [274]. EEG source imaging was further used offline to localize and image cortical activation during MI tasks performed during online BCI experiments where the online decoding was based on sensor data only [110], [111], [112]. This approach was also implemented in real-time online MI BCI experiments for continuous cursor and robotic arm control [164], [165], where the online decoding was based on estimated source signals. To perform EEG source imaging, an inverse transform such as minimum norm estimate (MNE) and low-resolution electromagnetic tomography (LORETA) is applied to convert EEG data recorded on the scalp (the sensor domain) into cortical source estimates (the source domain). Second, a region of interest in the brain cortex is selected according to the specific task or by a data-driven approach to realize an adaptive selection [275]. Then, the data can be treated according to traditional decoding methods or deep learning methods [276].

Source domain analysis is well suited for deciphering the precise movements of distinct arm segments. It further enables the decoding of fine MI movements [166] and realizes motor control for a robotic arm through the interpretation of MI tasks. While the computational demands of this approach can be high, source analysis has been successfully deployed in online MI BCI applications [164], [165].

#### 6) BCI Decoding Methods Outlook:

Presently, decoding methods prioritize three main goals: elevating classification accuracy, establishing calibration-free BCIs, and enhancing the robustness of BCIs. The need for higher classification accuracy is evident, as it facilitates effective communication, precise control of external devices, and an improved user experience. Calibration-free BCIs contribute to a more user-friendly and efficient experience, particularly crucial for real-world applications where users can swiftly engage with the system without undergoing lengthy calibration sessions. Robust BCIs address session-to-session non-stationarity and inter-subject variability, ensuring consistent performance across sessions and subjects, thereby enhancing overall BCI effectiveness.

While the decoding methods are categorized into distinct sections, it is important to note that these divisions are not strictly separated. Rather, there is often interconnectivity and overlap between these categories. For instance, transfer learning may be equipped with a CNN for intra-subject classification and using a strategy by adaptive learning to realize inter-subject transfer. Compared with offline applications, online evaluations provide a more realistic and dynamic assessment of a decoding method’s performance in practical, real-world scenarios. While current decoding methods in BCI research predominantly undergo offline testing using pre-recorded datasets, there is a crucial need to encourage and prioritize online applications, especially for emerging machine learning-based methods. In fact, a recent study implementing EEGNet and PointNet demonstrates the merits of deep learning for continuous tracking of a virtual object using MI paradigms in an online setting [221].

## V. BCI Software

As the previous sections demonstrate, there is now ample evidence that non-invasive BCI systems can be used, together with appropriate feedback/conditioning protocols, to replace, restore, improve, enhance, or supplement functions lost due to different neurological disorders [1], [277]. The realization of these possibilities crucially hinges on the effective and efficient implementation and validation of various BCI approaches. Unfortunately, the implementation of any new BCI system involves complex technology and the result may need to support different situations.

BCI systems are technically complex; they acquire signals from the brain and possibly other physiological or behavioral sources, analyze them to produce output commands, and finally realize the output and associated feedback. The acquisition of these signals is accomplished using specialized hardware and requires the implementation of proprietary software interfaces to configure the device and acquire data simultaneously. As an additional complication, the acquisition of all signals often needs to be synchronized. This is technically challenging as different sampling rates and acquisition delays must be accounted for across the different input devices. Finally, all of the involved device interactions and processing functions must occur in real time, with minimal delays and consistent timing. While there is no formal definition of these timing requirements, it is clear that feedback must be perceived as continuous (e.g., must be provided >20 times per second). Thus, the BCI software has less than 50 ms to store, visualize, and process the acquired data, and to produce all necessary outputs. These timing requirements necessitate highly optimized coding that minimizes CPU load and latency variation that may be introduced by the operating system or other factors. Implementing and integrating these functions is challenging, time-consuming, and costly.

Large multi-center BCI research or development efforts create additional challenges. These efforts usually involve different personnel who conduct experiments or analyze data in different locations. Thus, such large BCI efforts require the creation of many different BCI systems that all must store the resulting data in a standard format that is properly annotated. This means that all experimental parameters and event markers must be included to make the data practical for analysis by a person who is not intimately familiar with all experimental and technical details of a particular BCI experiment. Thus, there is an important need to facilitate such large BCI research and development efforts that vary in technical or neuroscientific approach, people, site, and are executed over long periods of time.

The following two sections summarize the existing toolboxes and BCI software platforms that can address these relatively simple or more complex needs, respectively (Table II).

### A. Toolboxes

Toolboxes relevant to BCI development provide functions that simplify the development of an individual BCI system. BCI toolboxes are usually developed on top of commercial high-level platforms (MATLAB, SIMULINK, or LabVIEW) or, more recently, using scripting languages such as Python.

MATLAB provides the basis for a number of BCI toolboxes for the presentation of behavioral paradigms (e.g., Psychophysics Toolbox [278]) and the post-hoc analysis and visualization of biosignals (e.g., EEGLab, [279]; BioSig [280]; Brainstorm [281]; FieldTrip [282]; and MNE [283]). EEGLab, BioSig and FieldTrip have been extended into toolboxes for rapidly prototyping and evaluating online BCIs, called BCILAB, rtsBCI and FieldTrip buffer, respectively.

SIMULINK provides the basis for g.BCIsys [284], a commercial SIMULINK-based toolbox that provides a front-end interface for rapid BCI research and development to users of g.tec hardware. LabVIEW has been the basis for several BCI demonstrations and has been used to implement the BCI toolbox Craniux [285]. The popularity of Python in the BCI community has increased over the past decade, and quite a few toolboxes have been implemented to facilitate BCI development. They include BciPy [286]; PyBCI [287]; MEDUSA [288]; Lab Streaming Layer [289]; pySPACE [290]; PsychoPy (for presentation of stimuli) [291]; and a set of toolboxes from the Berlin BCI group: Pyff – a framework for feedback applications and stimulus presentation [292]; Mushu – a toolbox for BCI signal acquisition [293]; and Wyrm – a BCI analysis toolbox [294].

### B. BCI Software Platforms

Just like BCI toolboxes, BCI software platforms offer functions that facilitate the development of BCI systems. To do this, they contain support for different signal acquisition hardware, signal processing and visualization routines, and different types of user feedback. They also have complete implementations of different BCI approaches that have been validated in different contexts, contain ample documentation, are maintained consistently to adapt to variations in a device’s software interface or operating systems, and provide a whole ecosystem of tools to interact online and offline with other tools or programming languages.

BCI software platforms are usually implemented in low-level programming languages (e.g., C++), compiled into native machine code, and executed without dependencies on commercial libraries. Mature platforms also support economical development and deployment across many laboratories and users. While many general-purpose BCI software platforms have been proposed, e.g., BCI++ [295], xBCI [296], TOBI [297], MindDesktop [298], and UniPA BCI [299], only BCI2000 [300], [301] and OpenViBE [302] have been consistently developed for many years, and have enjoyed widespread adoption across many laboratories.

#### 1) OpenViBE:

The OpenViBE software platform has been developed since 2007 [302]. OpenViBE is implemented in C++ and can be compiled on Windows and Linux; binaries are provided for Windows. OpenViBE is based on an architecture that facilitates the integration, expansion, and configuration of modular functionality, while the graphical interface makes OpenViBE easy to use for a wide range of investigators, including engineers, scientists, and clinicians. These two factors make OpenViBE well-suited to supporting the implementation of different BCI approaches.

#### 2) BCI2000:

The BCI2000 software platform has been developed since 1998 [300], [301]. BCI2000 is implemented in C++ and can be compiled on Windows and Linux; binaries are provided for Windows. Its implementation is based on a model that can describe any BCI system [300], [303]. In accordance with this model, BCI2000 has four modules that communicate with one another: Source (data acquisition and storage); Signal Processing; User Application; and Operator interface. The modules communicate through a documented network-capable protocol based on TCP/IP. The implementation of BCI2000 is highly optimized, so that it can support even very demanding BCI configurations with good timing characteristics [304].

BCI2000 provides interfaces to Matlab, SIMULINK, LabView, and Python. It has extensive scripting functionality that can be used on the command line using various approaches that include Matlab, Python, through a Telnet interface, or with a Windows dynamic link library (DLL). With these capabilities, BCI2000 can be used to implement applications with a completely custom graphical interface.

The BCI2000 data storage format accommodates variations in the digitized signals (e.g., in number of channels, sampling rate), defines the operating protocol, and includes a record of all events (e.g., feedback to user, device control, artifact detection) that occur during operation. BCI2000 has a roster of existing implementations with primarily technical documentation. These implementations can realize different BCI designs and readily usable methods, and employ commercially available and relatively inexpensive hardware components. BCI2000 is described in detail in a book [301], as well as in multiple book chapters [305], [306], [307], [308], [309], [310], [311] and peer-reviewed articles [300], [304], [312].

### C. Considerations for Designing BCI Software

The initial and important choice for designing BCI software is whether to implement it from scratch using a particular programming language, or whether to proceed using the existing toolboxes or BCI software platforms described above in order to reduce the time, complexity, and cost associated with BCI software development [313].

Implementing BCI software from scratch can be appropriate (and is only practical) if the technical demands are relatively low, such as, for example, in a student project that aims to use a consumer BCI headset to create simple visualizations/feedback of a particular brain signal. It may also be the best choice for clinical BCI systems that need to pass regulatory approval and whose functionality is relatively fixed.

Using BCI toolboxes can be appropriate when the technical demands are somewhat more complex but when there is an expectation that BCI software design, data collection, and data analysis are performed by the same person. Example situations are the development of a BCI system to support one research study by an individual graduate student.

Using BCI software platforms is most appropriate when the technical demands are complex (such as the simultaneous use of different hardware), and when there is an expectation that over the course of several years, many different BCI systems are needed to support diverse experimentation executed by different people in potentially different locations.

### D. Impact of BCI Software

The ultimate purpose of BCI software is to facilitate the implementation and testing of different BCI approaches. Thus, the success of BCI software in achieving this purpose can be measured by the number of scientific studies that they have supported. The illustration in Fig. S2 indicates the number of publications that have been supported by different BCI software packages. The benefit of the use of BCI software platforms is an improvement in practicality or cost associated with BCI system development [313].

## VI. Conclusion and Outlook

This review demonstrates that the field of non-invasive BCI research is active and prolific. The substantial progress in BCI research and the increasing availability of affordable and capable hardware, software, and signal processing/AI components have attracted an increasing number of scientists, engineers, and clinicians to participate in the field. Current research incorporates a broad range of neural signals that can be detected non-invasively, various classes of modern decoding algorithms, and different types of output devices such as robotic arms, motor neuroprosthetics, wheelchairs, or spellers. It also successfully transfers and adapts knowledge from other areas such as cognitive neuroscience (e.g., user training) or AI (e.g., sequence-to-sequence learning). Game- or meditation-based training protocols that target a particular mental state can also be combined with virtual and augmented reality technologies to facilitate a more cooperative communication between brain and machine. Despite these creative approaches, BCI illiteracy persists in some participants, calling for further innovations in neuroscientific protocol design and ML/AI algorithms. Moreover, AI algorithms, computer vision techniques, and increasing computer power continue to push the limits of neural decoding and help adapt various BCI applications to scenarios that better represent daily life.

There is no doubt that the field of non-invasive BCI research has made substantive progress in broadening its scientific and technical approaches and is now a mature research enterprise that has produced BCI demonstrations across the world. As the general public has become aware of this increasingly common accomplishment with healthy individuals, commercial technology companies have also become interested in further developing BCI systems. Indeed, companies such as Neuralink, Synchron and Meta have recently invested in similar technologies that may also allow healthy users and various patient populations to one day augment their lives [314], [315]. Ideally, further placing BCI technology in the public eye in this way may open new opportunities for larger collective innovation across the field. As such, additional resources would be greatly beneficial to address various technical problems that will certainly continue to occupy the attention of the BCI community for years to come. These issues include, but are not limited to the construction of deep learning models that can better extract and decode mental intention signals buried within noisy EEG, the development of robust wireless hardware for easy BCI experimentation in naturalistic settings, practical electrodes that can be used without gel but have a research-like impedance, the generalization of BCIs across subjects or tasks, the appropriate integration of large language models, the elimination of eye-blink or ambulation-based artifacts, and the development of more capable and versatile BCI software.

It is evident that publications and participant numbers used to demonstrate and validate technical advances far outnumber those applying such progress to patient populations. This is somewhat counterintuitive as the non-invasive nature of these systems should help facilitate more widespread clinical translation and acceptance. Such phenomena may be a result of the high level of heterogeneity across individual patients and conditions, relatively small effect sizes for neuromuscular rehabilitation metrics, and/or patient compliance. It may also be due to the lack of robust decoding techniques. In the future, integrating BCI technology with on-demand and closed-loop neuromodulation technologies, such as transcranial focused ultrasound stimulation, transcranial electric stimulation, or transcranial magnetic stimulation could enhance feedback directed at the brain and increase the intended effects while further lowering the effort and burden placed on users. The integration of neuromodulation with brain decoding may help bring BCI technology into the clinic for patients that require further facilitation of communication [27] or better control of a computer/robotic device [26], and expand its applications to patient populations extending beyond the neuromuscular realm, such as those suffering from neuropsychiatric disorders [316].

In summary, the field of non-invasive BCI research has witnessed substantial progress over the past decade. Continued research efforts on neuroscientific paradigms, ML/AI algorithm innovation, and the increased attention on BCI by the scientific community and larger society will further stimulate and facilitate research progress on non-invasive BCI. Future research shall be directed at innovating fundamental BCI technology by leveraging recent technological breakthroughs such as AI, improving individual system components and producing technical demonstrations, and by identifying and solving pressing problems of patients and clinicians.

## Supplementary Material

supp1-3449790

## Figures and Tables

**TABLE I:** Summary of Current Advanced Decoding Methods for EEG-based BCI

Decoding method	Algorithm	Literature
Deep learning	CNN	[210]-[216]
	Graph based methods	[217]-[221]
	LSTM	[222],[223]
	Neural networks with attention mechanisms	[224]-[226]
	Transformer	[227]-[229], [233]
	Hybrid deep learning	[230]-[233]
Transfer learning	Fine-tune	[230],[236]-[242]
	Domain adaptation	[244]-[246]
	Domain adversarial	[247]-[251]
	Few-shot learning	[252]-[257]
Manifold-based classification	Riemannian manifold	[258]-[261]
	Kernel-based nonlinear manifold	[262]
	Grassmann manifold	[263]
Adaptive learning	Supervised learning	[238]
	Unsupervised learning	[264],[265]
	Semi-supervised learning	[266]-[268]
EEG source analysis	EEG source imaging	[110]-[112], [121],[164]-[166],[270]-[276]

**TABLE II:** Overview of BCI Toolboxes and Software Platforms

	Language	License	Website
*Toolboxes*			
Psychophysics	Matlab	MIT	http://psychtoolbox.org
EEGLab	Matlab	GPL GNU	http://sccn.ucsd.edu/eeglab
BioSig	Matlab	GPL GNU	http://biosig.sourceforge.net
Brainstorm	Matlab	GPL GNU	http://neuroimage.usc.edu/brainstorm
FieldTrip	Matlab	GPL GNU	http://www.fieldtriptoolbox.org
MNE	Matlab	BSD	http://martinos.org/mne
BCILAB	Matlab	GPL GNU	http://sccn.ucsd.edu/wiki/BCILAB
rtsBCI	Matlab	GPL GNU	http://biosig.sourceforge.net
FieldTrip buffer	C++	GPL GNU / BSD	https://www.fieldtriptoolbox.org/development/realtime/buffer/
g.BCIsys	Simulink	commercial	https://www.gtec.at/product/bcisystem/
Craniux	Lab View	?	?
BciPy	Python	Hippocratic License	https://github.com/CAMBI-tech/BciPy
pybci	Python	MIT	https://github.com/LMBooth/pybci
MEDUSA	Python	Creative Commons	https://www.medusabci.com
Lab Streaming Layer	Python	MIT	https://github.com/sccn/labstreaminglayer
pySPACE	Python	GPL GNU	https://pyspace.github.io/pyspace/
PsychoPy	Python	GPL GNU	http://www.psychopy.org
pyff	Python	GPL GNU	https://github.com/bbci/pyff
mushu	Python	GPL GNU	https://github.com/bbci/mushu
wyrm	Python	MIT	https://github.com/bbci/wyrm
*BCI Software Platforms*			
BCI++	C++	GPL GNU	?
xBCI	C++	?	https://xbci.sourceforge.net (outdated)
TOBI	C++	LGPL	https://github.com/tools4BCI/
MindDesktop	?	?	https://www.oriossmy.com/project01
UniPA BCI	C++	GPL GNU	https://github.com/slabua/AugmentedBCIFramework
BCI2000	C++	GPL GNU	http://www.bci2000.org
OpenViBE	C++	GPL Affero	http://openvibe.inria.fr
