# Supplementary material for: Non-Invasive Brain-Computer Interfaces: State of the Art and Trends
Source: IEEE Rev Biomed Eng. Author manuscript; Available in PMC 2025 Mar 4. (PMC11861396; doi:10.1109/RBME.2024.3449790)
Supplement: supp1-3449790 [file NIHMS2052501-supplement-supp1-3449790.pdf]

Supplementary Materials

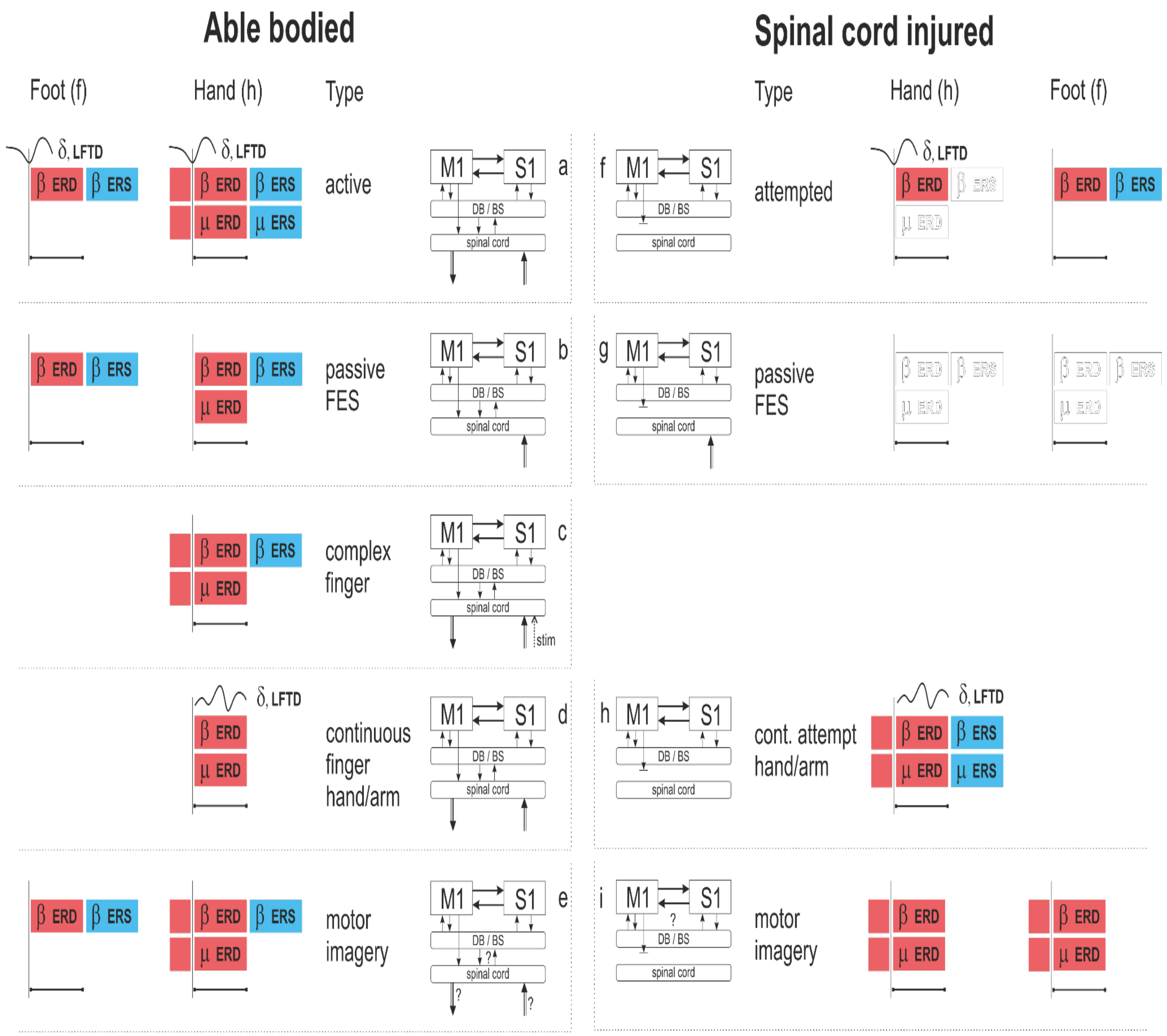

Fig. S1. Overview of ERD/ERS pattern before, during and after specific types of movement in able-bodied (a-e) and individuals with spinal cord injury (f-i). Assuming a typical trial structure, the red boxes indicate periods of task performance and blue boxes indicate post-task periods. The corresponding neuro-structural models are proposed explanations for each movement type and in some cases specific pathways (arrows) have not been fully proven. These instances are marked with a "?".

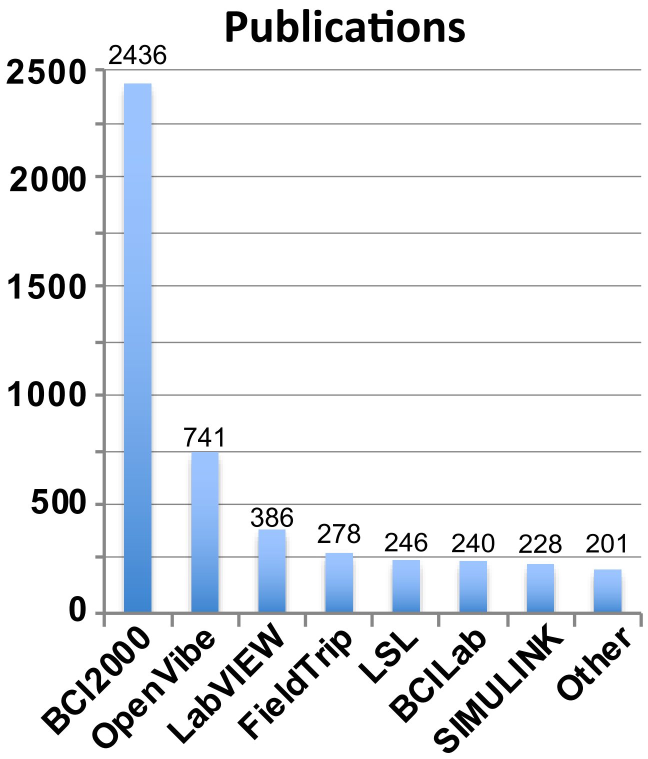

Fig. S2. Scientific publications using various BCI software.
